# Macrophage Control of Phagocytosed Mycobacteria Is Increased by Factors Secreted by Alveolar Epithelial Cells through Nitric Oxide Independent Mechanisms

**DOI:** 10.1371/journal.pone.0103411

**Published:** 2014-08-04

**Authors:** Dagbjort H. Petursdottir, Olga D. Chuquimia, Raphaela Freidl, Carmen Fernández

**Affiliations:** Department of Molecular Biosciences, The Wenner-Gren Institute, Stockholm University, Stockholm, Sweden; Bose Institute, India

## Abstract

Tissue-resident macrophages are heterogeneous with tissue-specific and niche-specific functions. Thus, simplified models of macrophage activation do not explain the extent of heterogeneity seen *in vivo*. We focus here on the respiratory tract and ask whether factors secreted by alveolar epithelial cells (AEC) can influence the functionality of resident pulmonary macrophages (PuM). We have previously reported that factors secreted by AEC increase control of intracellular growth of BCG in macrophages. In the current study, we also aimed to investigate possible mechanisms by which AEC-derived factors increase intracellular control of BCG in both primary murine interstitial macrophages, and bone marrow-derived macrophages and characterize further the effect of these factors on macrophage differentiation. We show that; a) in contrast to other macrophage types, IFN-γ did not increase intracellular growth control of *Mycobacterium bovis*, Bacillus Calmette-Guérin (BCG) by interstitial pulmonary macrophages although the same macrophages could be activated by factors secreted by AEC; b) the lack of response of pulmonary macrophages to IFN-γ was apparently regulated by suppressor of cytokine signaling (SOCS)1; c) AEC-derived factors did not induce pro-inflammatory pathways induced by IFN-γ e.g. expression of inducible nitric oxide synthase (iNOS), secretion of nitric oxide (NO), or IL-12, d) in contrast to IFN-γ, intracellular bacterial destruction induced by AEC-derived factors was not dependent on iNOS transcription and NO production. Collectively, our data show that PuM were restricted in inflammatory responses mediated by IFN-γ through SOCS1 and that factors secreted by AEC- enhanced the microbicidal capacities of macrophages by iNOS independent mechanisms.

## Introduction

Macrophages are essential elements in both innate and adaptive immunity playing a crucial role in host defense. In addition to being a first line of resistance against pathogens, macrophages contribute to remodeling and repair. Tissue-resident macrophages are a heterogeneous population of immune cells that fulfill tissue-specific and niche-specific functions. These range from dedicated homeostatic functions, such as clearance of cellular debris and iron processing, to central roles in tissue immune surveillance, response to infection and the resolution of inflammation. Understanding the mechanisms that dictate tissue macrophage heterogeneity should explain why simplified models of macrophage activation do not explain the extent of heterogeneity seen *in vivo*.

In the lung tissue, although a number of immune cells secrete many of these factors, alveolar epithelial cells (AEC) and other non-immune cells are also able to produce a broad variety of factors including cytokines and chemokines that affect inflammatory responses in macrophages [1]–[6]. We have reported that factors secreted by AEC can influence both the phenotype and other characteristics of resident pulmonary macrophages (PuM) [7], confirming that the micro-environment provided by cytokines and other factors contribute to macrophage polarization and hence to their antimicrobial properties.

Polarized macrophages have been broadly classified into two groups: M1 (classically activated macrophages) or M2 (alternatively activated macrophages) [8]. M1 macrophages respond to type 1 inflammatory cytokines and microbial products such as lipopolysaccharide (LPS) to active inducible nitric oxide synthase (iNOS) and the production of reactive oxygen species (ROS) [8]–[10]. Thus, M1 cells are thought to represent a “common host response” for microbicidal capacities [8]–[10] while M2 cells are immunoregulatory with poor microbicidal activity [8]. Since M2 macrophages display a very broad functional spectrum, a further subcategorization has been proposed based on the *in vitro* stimuli used for the generation of the different phenotypes, where culturing with IL-4 or IL-13 yields M2a; induced by immune complexes, TLR agonists or IL-1R yield M2b; IL-10 or glucocorticoids yield M2c [11].

Previously, we have shown that treatment of PuM with AEC-derived factors affect their functional properties seen e.g. in morphological alterations demonstrated by elongated cell shape as well as increased expression of the marker macrophage mannose receptor (MMR), both features indicating M2 polarization [7], [12], [13]. However, treatment with AEC-derived factors also increases the PuM phagocytic activity as well as their capacity to control mycobacteria intracellular growth upon infection with *Mycobacterium bovis*, Bacillus Calmette-Guérin (BCG), characteristics more related to M1 polarization [7].

In the present study we investigated possible mechanisms for the induction of intracellular bacterial growth control in macrophages mediated by AEC-derived factors and compared this with treatment with IFN-γ, a classical inducer of M1 activation. Our data suggested that AEC-derived factors induced microbicidal functions through iNOS-independent mechanisms and that suppressor of cytokine signaling 1 (SOCS1), a known inhibitor of IFN-γ signaling, [14]was involved in the inability of PuM to respond to IFN-γ.

## Materials and Methods

### Mice

All mouse experiments were approved and performed in accordance with the guidelines of the Animal Research Ethics Board at Stockholm University (ethical approval ID: N463/12). The studies were performed using 8–12-week-old female C57BL/6 mice purchased from NOVA-SCB, Sweden. All animals were kept at the Animal Department of the Arrhenius Laboratories, Stockholm University, Sweden and housed in pathogen-free conditions. Mice were supervised daily and sentinel mice were used to assess and ensure pathogen free conditions in the facility. IFN-γ^-/-^SOCS1^-/-^ mice were kindly provided by M. Rottenberg (MTC, Karolinska Institutet, Stockholm, Sweden) and experiments conducted in accordance with the guidelines of Karolinska Institutet.

### Bacteria

We used BCG, transformed with a dual reporter plasmid containing the human codon-optimised and fluorescence-enhanced EGFP and the bacterial luciferase A and B (luxAB) genes from *Vibrio harveyi* [15] herein called GFP-BCG. Bacterial contents are expressed as relative luminescence units (RLU). The increase of OD and RLU was followed for all batches and experiments. All batches were also routinely controlled for CFU and RLU values. The correlation between CFU and RLU has been previously published as supplementary data [16], [17]. Furthermore, a correlation between CFU and RLU has even been confirmed in the experimental setup used in this study, i.e. in an assay determining intracellular survival of bacteria within macrophages (unpublished data). GFP-BCG was grown in Middlebrook 7H9 broth (DIFCO, Sparks, MD, USA) supplemented with albumin-dextrose-catalase (ADC), 0.5% glycerol, 0.05% Tween 80 (vol/vol), and 50 µg/ml hygromycin for 10–15 days. Bacteria were collected at a log phase of growth (absorbance 1.0 measured at OD_650_) and frozen in PBS with 10% glycerol and kept at −80°C. Before infection of cell cultures, a vial was thawed and placed in culture as above for 4–5 days, reaching an early log phase (OD_650_ ∼0.3) corresponding to approximately 3×10^8^ CFU per ml. To determine the RLU, Decanal (0.01% v/v in ethanol) (Sigma-Aldrich) was used as a specific substrate for the bacterial enzyme luxAB. The samples were mixed immediately and luminescence measured after 15 sec in a Modulus, Turner Bio Systems luminometer.

### Isolation of alveolar epithelial cells (AEC) and interstitial pulmonary macrophages (PuM)

Total pulmonary cells were prepared using Corti's protocol [18] with previously described modifications [16], [17]. PuM were isolated from either C57BL/6 or IFN-γ^-/-^SOCS1^-/-^ mice [19] by positively selecting CD45^+^ cells from total pulmonary cells using MACS (Miltenyi Biotec, Bergish Gladbach, Germany) and a subsequent adhesion isolation. After isolation, cells were cultured for 48 h in RPMI (Gibco-Invitrogen, Paisley, UK) supplemented with 10% FCS, 2 mM L-glutamine, 100 U/ml penicillin, 100 µg/ml streptomycin, 0.02 M Hepes, and 0.05 M 2-mercaptoethanol (Sigma) (complete RPMI) at 37°C and 5% CO_2_. Next, cells were washed to remove non-adherent cells and debris. In average, 98% of the cells were positive for the macrophage marker F4/80, as determined by flow cytometry. AEC were obtained by depleting CD45^+^ and CD146^+^ cells from lung cell preparations using MACS. In average, 92-95% of these CD45^-^CD146^-^ cells exhibited an AEC phenotype, where approximately 22% expressed podoplanin (type I AEC) and approximately 72% expressed CD74 (type II AEC) as determined by flow cytometry. Cell viability was determined by trypan blue exclusion to be over 90%.

For the preparation of AEC-derived media, AEC were plated (5×10^4^ cells/well) in 96-well flat bottom plates (Costar, NY, USA) and cultured for 48 h. After washing the cells three times, cells were again kept unstimulated for 24 h. These cell culture supernatants were collected, pooled, filtered through a 0.2 µm filter and stored at −70°C until use. These cell culture supernatants were named AEC_sup_.

### Isolation of alveolar macrophages (AM) and peritoneal exudate macrophages (PEM)

AM were isolated from bronchoalveolar lavage (BAL) using a previously described protocol [20] with some modifications. Briefly, a bronchoalveolar lavage (BAL) was performed with 1–2 ml of pre-warmed (37°C) PBS-0.5 mM EDTA (Gibco) via the trachea 3-4 times. After lavage, BAL fluids were pooled and put on ice. PEM were isolated as previously described [20]. The mouse's skin was removed carefully leaving the peritoneal membrane intact. The peritoneal cavity was rinsed twice with RPMI (5 ml) and the collected peritoneal exudate cells were pooled and placed on ice. BAL and peritoneal exudate cells (PEC) were then washed twice in ice-cold RPMI and plated in 48-well flat bottom plates (Costar, NY, USA) at a cell concentration of 2×10^5^ cells/well (BAL) and 1.5×10^6^ cells/well (PEC) for 2 h in complete RPMI. Cells were washed 2–3 times to eliminate debris and non-adherent cells and cultured in complete RPMI overnight. After 24 h, ∼98 ± 1% of BAL cells and 98 ± 1% of PEC were positive for the macrophage marker F4/80 and were considered as AM and PEM, respectively. A further characterization showed that, 83 ± 8% of AM were F4/80^+^ CD11c^+^, a typical AM phenotype, 15 ± 3% were F4/80^+^CD11b^+^, and 8±1% were F4/80^+^CD11b^+^CD11c^+^ whereas approximately 99 ± 1% of PEM were F4/80^+^ CD11b^+^.

### Generation of bone marrow derived macrophages (BMM)

BMM were harvested and frozen as previously described [21]. After thawing, the bone marrow cells were cultured in complete RPMI supplemented with 20% L929 cell conditioned medium (as a source of macrophage colony-stimulating factor) and cultured for 6–7 days, replacing medium every second day. Before use, growth factor-containing media were removed and the BMM cultured in complete RPMI for 24 h. BMM from caspase 1^-/-^ mice were kindly provided by S. Musciol and B. Henriques-Normark (MTC, Karolinska Institutet, Solna, Sweden)

### Infection and intracellular bacterial growth

BMM, PuM, AM and PEM were cultured in 48 or 12-well plates as described above 1×10^5^ and 4×10^5^ cells per well respectively. Cells were kept with complete RPMI without antibiotics for 24 h prior to infection with GFP-BCG at a MOI 10∶1 (bacteria:cell) for 4 h. After infection, cells were washed three times and treated with gentamicin (100 µg/ml) for 30–60 min at 37°C. After further washing, cells were either kept untreated or treated with 20 ng/ml of IFN-γ (Mabtech, Sweden), or IL-1β (1 or 10 ng/ml) or AEC_sup_ (diluted 1∶2 in cell culture media). For inhibition of iNOS 1 mM of N^G^-monomethyl-L-arginine (NMMLA) (Sigma) was added together with either IFN-γ or AEC_sup_. For reducing reactive oxygen species (ROS) in the cultures, cells were incubated with the ROS scavengers superoxide dismutase (SOD, 15 U/ml, Sigma-Aldrich) and catalase 500 µg/ml (Sigma-Aldrich). The bacterial loads were measured by determining RLU and data is displayed as RLU/10^6^ cells.

### Real- time quantitative PCR

Total RNA was harvested from BMM or PuM untreated or treated with either IFN-γ or AEC_sup_ at 4 or 24 h after the end of BCG infection using QIAzol Lysis Reagent (Qiagen). After isolation, the RNA was run on a 1% agarose gel to verify that the RNA was intact. RT-PCR was performed on 1 µg of RNA using SuperScript III Reverse Transcriptase (Invitrogen) with Oligo(dT)_20_ Primers (Invitrogen). Transcripts were quantified on a RotorGene 6000 real-time PCR cycler (Corbett Life Sciences) using Kapa Sybrfast qPCR Kit (Kapa Biosystems). Primer sequences used were: iNOS sense: CAGCTCGGCTGTACAAACCTT, antisense: CATTGGAAGTGAAGCGTTTCG, IL-1β sense: TGGTGTGTGACGTTCCCATT antisense: CAGCACGAGGCTTTTTTGTTG, Arg-1 sense: GCAGTGGCTTTAACCTTGGC, antisense: TGGCGCATTCACAGTCACTT. HPRT sense CCC AGC GTC GTG ATT AGC, antisense GGA ATA AAC ACT TTT TCC AAA TCC was used as a control gene and used to calculate dCt values. To determine the effect of treatment with IFN-γ or AEC_sup_, values were normalized to that of cells infected with BCG receiving no treatment and the fold induction (RQ) calculated.

### Cytokine and nitric oxide (NO) determinations

Cytokines and nitrite (an indirect indicator of NO production) were measured in cell culture supernatants from BMM and PuM un-treated or treated with either IFN-γ or AEC_sup_ at 4, 24 and 48 h after the end of infection with BCG. For ELISA determinations, the commercially, TNF, IL-6, CXCL10/Interferon gamma-induced protein 10 kDa (IP-10), IL-1β (R&D Systems) and IL-12, (Mabtech) were used to determine the cytokine levels in the culture supernatants according to the manufacturer's recommendations. The enzyme-substrate reaction was developed using p-nitrophenyl phosphate (Sigma) for IL-12 and tetramethylbenzidine substrate (R&D Systems) for the rest of determinations. Depending on the substrate used, the optical density was measured in a multiscan ELISA reader (Anthos Labtech Instruments, Salzburg, Austria) at 405 or 450 nm. NO production was determined by measuring nitrite concentration using the Griess reaction according to the manufacturers protocol (Sigma).

### Analysis of ROS production

BMM were prepared as described above and then pretreated with either IFN-γ (20 ng/ml) or AEC_sup_ (diluted 1∶2 in cell culture medium) for 24 h. Cells were then infected with BCG (MOI 1∶10) and 500 µM luminol (Sigma) added to the cultures. Plates were then placed in an EnSpire Multimode Plate Reader (PerkinElmer) and luminescence monitored at 37°C every 3 min for 15 h.

### Statistical analysis

Data are presented as the mean ± SD. Differences between treatments in the groups were analyzed using one-way ANOVA followed by Bonferroni's post-test for multiple comparisons. For qPCR data differences between treatment at different time-points were analyzed using a non-parametric one-way ANOVA (Kruskal-Wallis) with Dunn's post-test. Differences were considered significant at *p*<0.05. All data were analyzed using the GraphPad InStat version 5.0 (GraphPad Software, San Diego, CA, USA).

## Results

### Impaired control of intracellular BCG growth by PuM upon IFN-γ treatment

**Figure 1:**
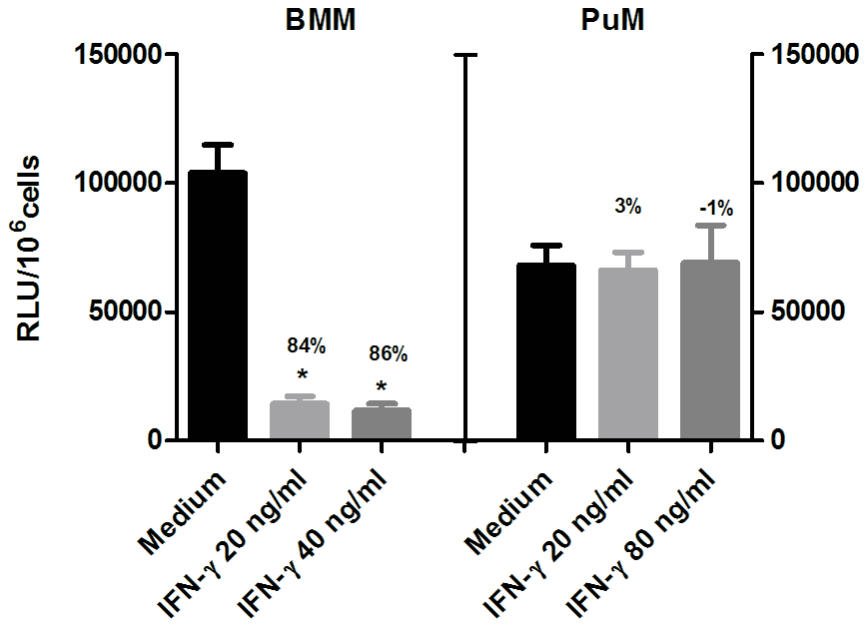
Impaired intracellular control of BCG growth by PuM upon IFN-γ treatment. BMM and PuM were infected with GFP-BCG. After infection, cells were left untreated or treated with IFN-γ (20, 40 and 80 ng/ml) for 48 h. Bacterial growth was evaluated by determining RLU. Data are shown as % reduction of phagocytosed bacteria evaluated as RLU. Values are means ± SD of the mean of a representative experiment from 3 independent experiments with 4 replicates each. The differences between groups of BMM and PuM were analyzed using a one-way ANOVA followed by Bonferroni‘s Multiple Comparison Test, * significantly different from medium control, P<0.05.

Since IFN-γ is known to mediate the microbicidal activation of macrophages against mycobacteria [22] we evaluated the response of PuM and BMM to IFN-γ. BMM and PuM were infected with GFP-BCG as described in Materials and Methods, treated with different concentrations of IFN-γ (20, 40 and 80 ng/ml) or left untreated. Bacterial loads were monitored by measuring RLU. As expected, BMM reduced the intracellular bacterial content upon treatment with IFN-γ (Figure 1). In contrast, PuM were not able to reduce bacterial load even after treatment with doses of IFN-γ as high as 80 ng/ml (Figure 1). We further evaluated whether PuM needed to be activated prior to mycobacterial infection since it has been described that IFN-γ “activated” macrophages could better control mycobacteria growth [23], [24]. For this, PuM were treated with IFN-γ prior infection and bacterial loads monitored. We found that PuM pre-treated with IFN-γ were still unable to control intracellular BCG growth (Fig S1).

### PuM are specifically unresponsive to IFN-γ in the control of intracellular bacterial growth

**Figure 2:**
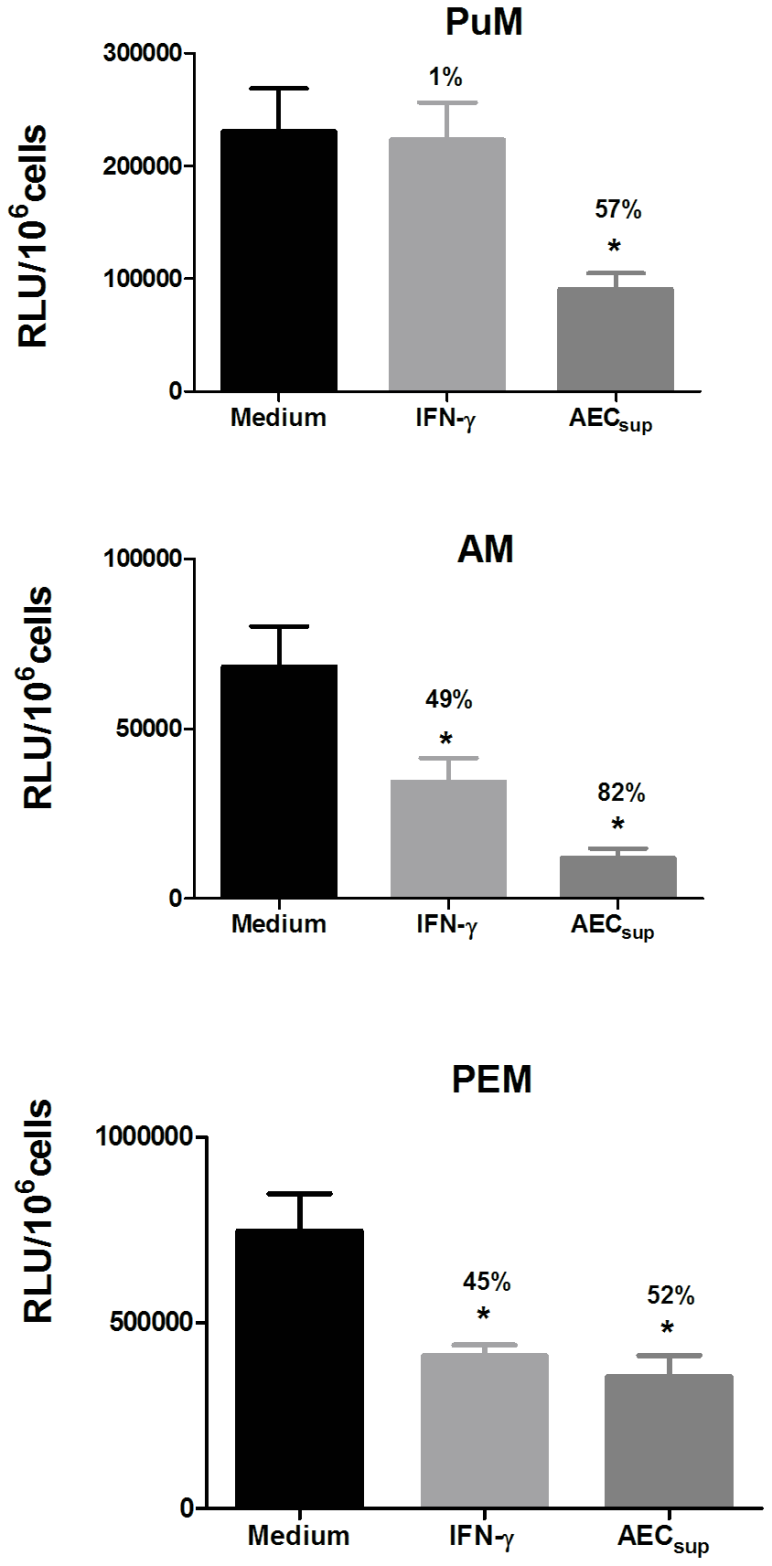
PuM are specifically unresponsive to IFN-γ in the control of intracellular bacteria. PuM, AM and PEM were infected with GFP-BCG. After infection, cells were treated with either IFN-γ or AEC_sup_ for 48 h or left untreated. Bacterial growth was evaluated by determining RLU. Data are shown as % reduction of phagocytosed bacteria evaluated as RLU. Values are means ± SD of the mean of 2 independent experiments with 4 replicates each. The differences between groups of PuM, AM and PEM were analyzed using a one-way ANOVA followed by Bonferroni‘s Multiple Comparison Test, * significantly different from medium control, P<0.05.

To understand whether the unresponsiveness to IFN-γ was a characteristic of pulmonary macrophages, we compared PuM with other tissue derived macrophages, namely AM and PEM obtained from lung and peritoneal exudates, respectively. Macrophages were treated with IFN-γ and with AEC-derived factors (AEC_sup_) since we have described previously [7] that treatment with AEC-derived media decreased intracellular bacterial loads in PuM. AM, PuM and PEM were isolated and infected with GFP-BCG and later, either left untreated or treated with IFN-γ or AEC_sup_. Bacterial loads were monitored by measuring RLU. In contrast to the effect on PuM, IFN-γ was able to induce a significant control of intracellular BCG growth in AM and PEM (49% and 45% reduction respectively) (Figure 2). Importantly, treatment with AEC_sup_ mediated reduction of intracellular BCG in the three types of primary macrophages, PuM, AM and PEM (Figure 2) indicating that PuM was specifically unable to respond to IFN-γ.

### IFN-γ and AEC_sup_ induce different cytokine profiles in PuM and BMM

**Figure 3:**
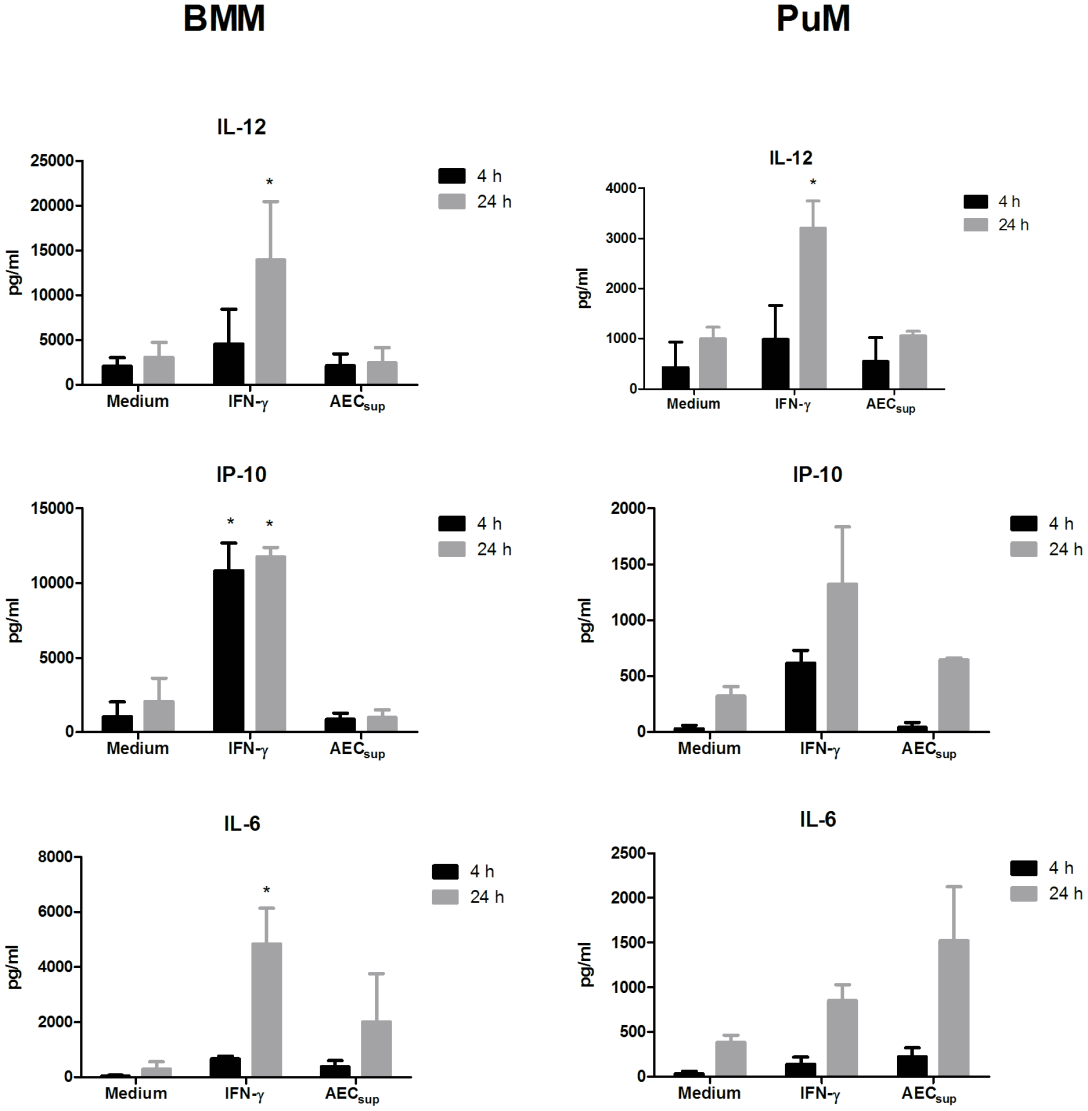
IFN-γ and AECsup induce different cytokine profiles in PuM and BMM. BMM and PuM were infected with GFP-BCG. After infection, cells were left untreated or treated with either IFN-γ or AEC_sup_. Cell culture supernatants were collected at 4 and 24 h after treatments. IL-12, IP-10 and IL-6, levels were measured using ELISA. Values are expressed as means ± SD, from 3 independent experiments. The differences between groups of PuM and BMM were analyzed using a one-way ANOVA followed by Bonferroni‘s Multiple Comparison Test, * Significantly different from medium control, p<0.05.

To further understand the different responses elicited in PuM and BMM by AEC_sup_ and IFN-γ, we stimulated the two cell types *in vitro* as described in Materials and Methods and collected supernatants 4 and 24 h later. We monitored the production of IL-12, IP-10 and IL-6 since these factors are associated with IFN-γ mediated signaling through the activation of receptor-associated JAKs. Two major observations are obvious from the data shown in Figure 3. Firstly, comparing the stimulation capacity of IFN-γ and AEC_sup_, the secretory profile was different in both cell types. AEC_sup_ did not induce production of IL-12 and IP-10 statistically above that of unstimulated cells but induced IL-6 secretion significantly in BMM at 4 h. Similarly, AEC_sup_ also tended to increase IL-6 secretion in PuMSecondly, although the profile of stimulation was similar, the secretion of all factors measured was lower in PuM compared with BMM.

### Impaired intracellular control of BCG by PuM upon IFN-γ treatment is regulated by SOCS1

**Figure 4:**
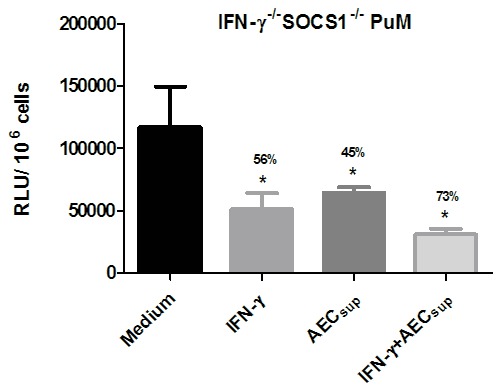
Impaired intracellular control of BCG growth by PuM upon IFN-γ treatment is regulated by SOCS1. PuM from IFN-γ^-/-^ SOCS1^-/-^ mice were infected with GFP-BCG. After infection, cells were left untreated or treated with IFN-γ, AEC_sup_ and IFN-γ + AEC_sup_ for 48 h. Bacterial growth was evaluated by determining RLU. Data are shown as % reduction of phagocytosed bacteria evaluated as RLU. Values are means ± SD of the mean of a representative experiment from 2 independent experiments with 4 replicates. The differences between groups of IFN-γ^-/-^ SOCS1^-/-^ PuM were analyzed using a one-way ANOVA followed by Bonferroni‘s Multiple Comparison Test. * significantly different from medium control, P<0.05.

To assess whether the reduced capacity of PuM to respond to IFN-γ was related to intracellular regulations of signaling, we tested this in PuM derived from IFN-γ^-/-^SOCS1^-/-^ mice. SOCS1 has been described to be a critical inhibitor of IFN-γ signaling [25] and also able to dampen early responses to BCG and *Mycobacterium tuberculosis* [19]. Thus, we evaluated the effects of IFN-γ and AEC_sup_ treatments on intracellular BCG control by PuM from IFN-γ^-/-^SOCS1^-/-^ mice. After IFN-γ treatment, PuM from IFN-γ^-/-^SOCS1^-/-^ mice controlled intracellular growth of BCG, to a similar extent as cells treated with AEC_sup,_ indicating that the lack of response to IFN-γ is likely not due to lack of surface IFN-γ receptor expression but rather that the response is under regulation of SOCS1 (Figure 4a). However, there were no differences seen in SOCS1 expression between BMM and PuM and both cell types upregulated SOCS1 upon stimulation with IFN-γ indicating that the selective lack of response to IFN-γ in PuM was not due to a lack of SOCS1 but rather an event downstream of SOCS1 (data not shown). Treating cells with both IFN-γ and AEC_sup_ tended to show an additive effect suggesting independent mechanisms of intracellular bacterial control (Figure 4a). Similarly to the results observed in the wild type mice, upon treatment with IFN-γ, the levels of IL-12 induced in PuM derived from IFN-γ^-/-^SOCS1^-/-^ mice were lower than in BMM (data not shown) Thus, reduced IL-12 secretion may not only be subjected to SOCS1 regulation but also to other mechanisms inherent to different tissue-derived macrophages.

### AEC_sup_ increases intracellular growth control without an effect on iNOS

**Figure 5:**
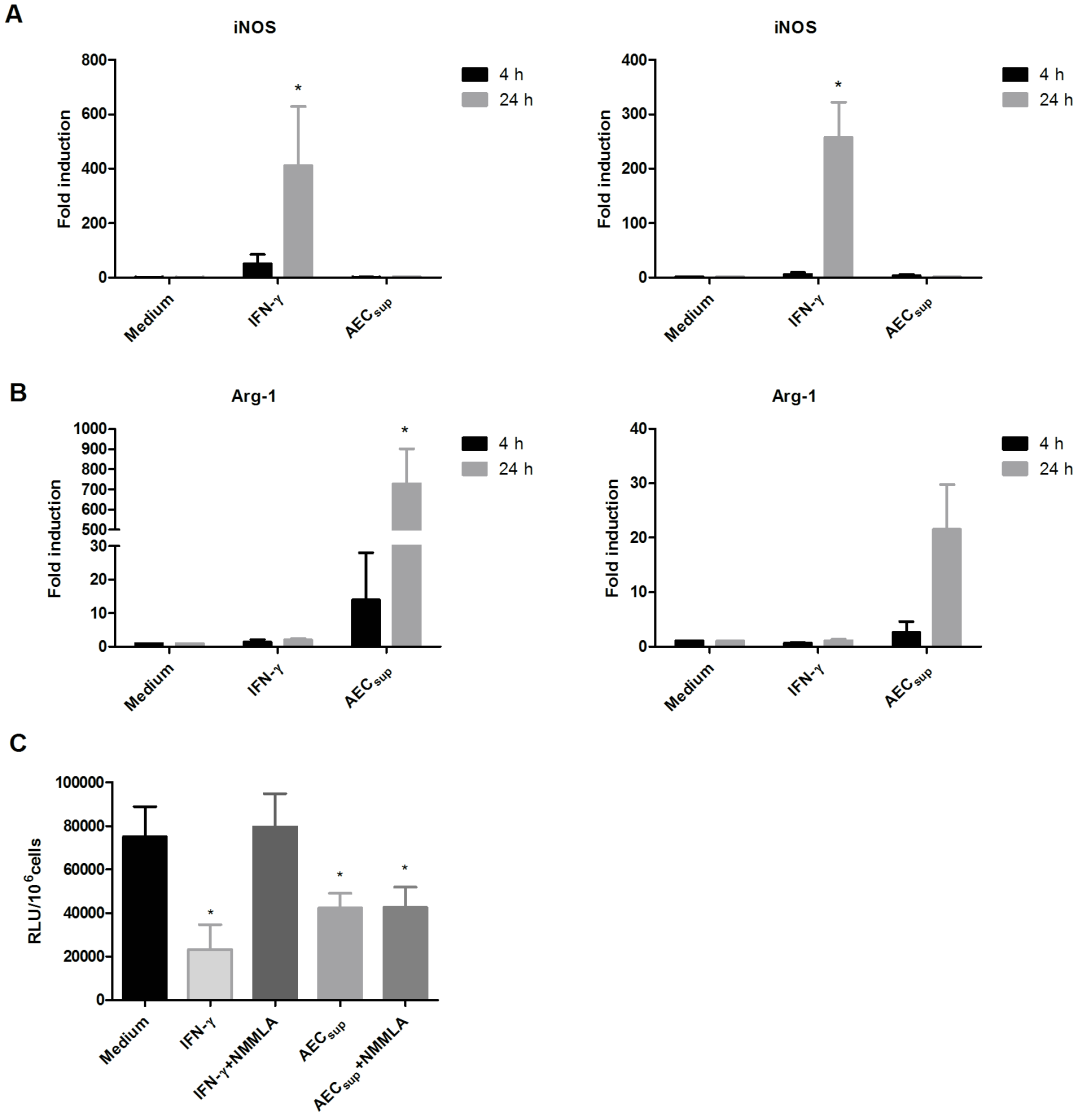
AEC_sup_ has opposite effects to IFN-γ on iNOS, and Arg-1 expression in BMM and PuM and mediates killing through an iNOS independent mechanism. BMM and PuM were infected with GFP-BCG. After infection, cells were treated with either IFN-γ or AEC_sup_ or 1 mM of NG-monomethyl-L-arginine (NMMLA) together with either IFN-γ or AEC_sup_ or left untreated. Total RNA was extracted from both cell-types after 4 and 24 h of treatment and the mean-fold accumulation of a) iNOS, b) Arg-1 ± SD from 3-4 experiments. In c) the effect of the iNOS inhibitor NMMLA on intracellular growth of BCG is shown. Bacterial growth was evaluated by determining RLU. Data are expressed as mean ± SD. The differences between groups of BMM and PuM were analyzed using non-parametric, one-way ANOVA (Kruskal-Wallis) with Dunn‘s post-test. * significantly different from medium control, P<0.05

Up-regulation of iNOS and the subsequent synthesis of reactive nitrogen species is currently believed to be one of the primary mechanisms downstream of IFN-γ-induced killing of mycobacteria [22]. On the other hand, Arg-1 can compete with iNOS for their common substrate L-arginine, and consequently, the expression of iNOS and Arg-1 is often reciprocally regulated in macrophages [26]. To reveal possible mechanisms for the observed intracellular bacterial control, we followed the expression of iNOS and Arg-1 induced in PuM and BMM upon treatment with AEC_sup_ or IFN-γ. We found that IFN-γ but not AEC_sup_ induced iNOS in both BMM and PuM (Figure 5a). In contrast, in both PuM and BMM, Arg-1 was induced only by AEC_sup_ and not by IFN-γ (Figure 5b). Notably, the levels of Arg-1 induced in BMM were higher than the levels induced in PuM. Addition of NMMLA, an inhibitor of iNOS, to cultures of BMM infected with BCG and treated with AEC_sup_ or IFN-γ, inhibited the bacterial load reduction mediated by IFN-γ but had no effect on AEC_sup_ activity (Figure 5c) confirming that AEC_sup_ increased intracellular killing using an iNOS independent pathway.

### AEC_sup_ induces IL-1β transcription and secretion

**Figure 6:**
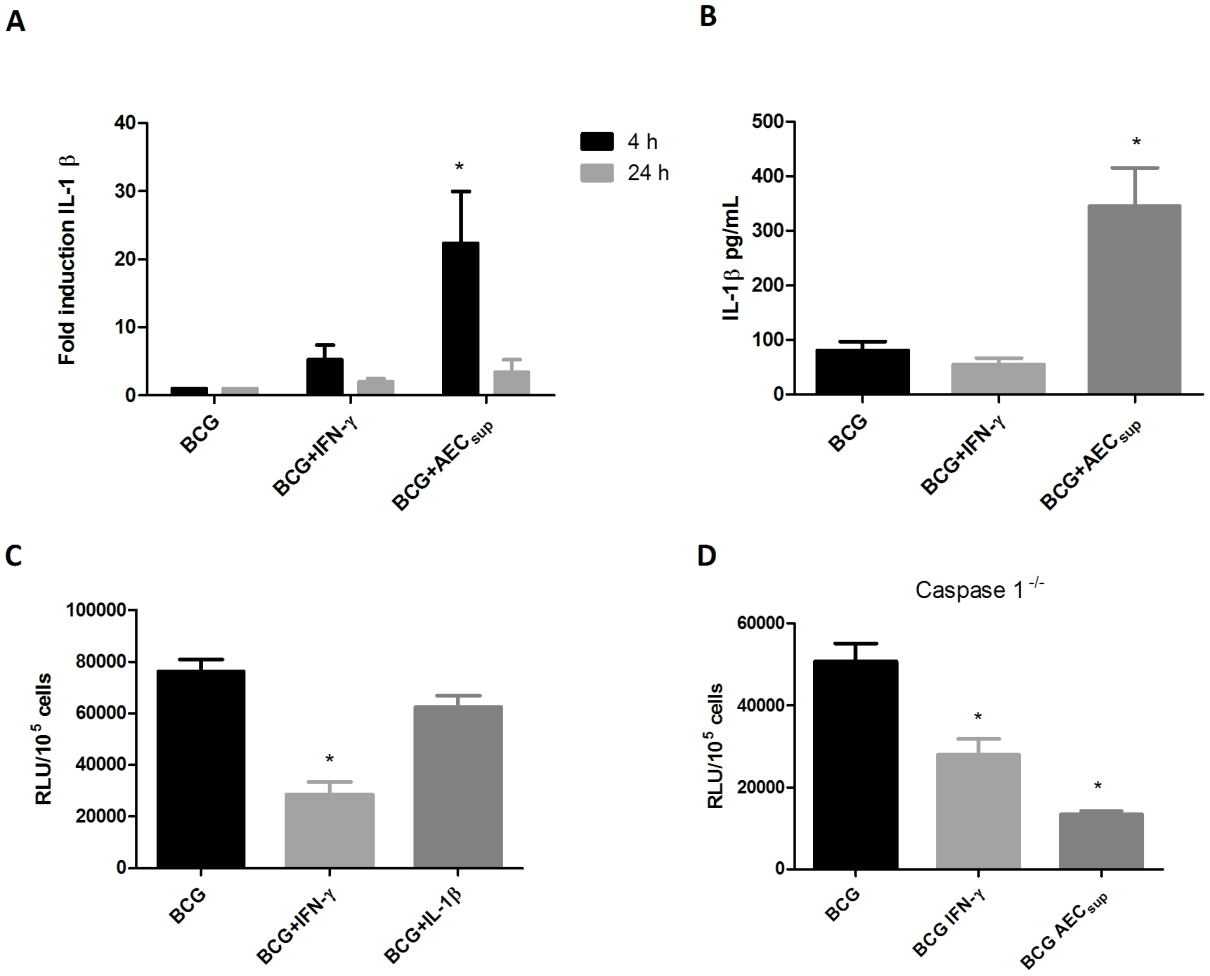
AEC_sup_ induces IL-1β expression and secretion without an effect of intracellular killing of BCG. BMM and PuM were infected with GFP-BCG. After infection, cells were treated with either IFN-γ or AEC_sup_ or left untreated. a) Total RNA was extracted from both cell-types after 4 and 24 h of treatment and the mean-fold accumulation of IL-1β transcripts shown. Data shows the average of 3 independent experiments. b) Cell culture supernatants were collected 24 h after infection and IL-1β measured with ELISA. Data shows the average of 6 independent experiments. c) The effect of incubating cells with IL-1β (1000 pg/ml) on intracellular growth of BCG. Data representative of 3 independent experiments d) The effect of AECs_up_ on intracellular growth of BCG in BMM from caspase1^-/-^ mice. Bacterial growth was evaluated by determining RLU. Values are expressed as means ± SD from 5 wells. The data is representative of 2 independent experiments. The differences between groups of BMM were analyzed using a one-way ANOVA followed by Bonferroni‘s Multiple Comparison Test. * significantly different from medium control, P<0.05.

IL-1β has been shown to be involved in the intracellular killing of mycobacteria through the induction of autophagy related mechanisms [27]. We therefore investigated whether AEC_sup_ affect IL-1β production. We observed a difference in the profile of induction of this interleukin by the two stimuli where only AEC_sup_ induced IL-1β transcription (Figure 6a) and secretion (Figure 6b) above that of cells only infected with BCG. We next tested whether this increase in IL-1β secretion in AEC_sup_-treated BMM might mediate the increased intracellular killing of BCG by these cells. However, addition of exogenous IL-1β to the cultures did not affect intracellular bacterial killing (Figure 6c). Finally, we asked the same question using BMM derived from caspase 1^-/-^ mice that are unable to secrete the mature form of IL-1β. We observed that AEC_sup_ activated BMM derived from caspase 1^-/-^ mice were able to control efficiently intracellular bacteria growth supporting the notion that IL-1β was not the key molecule in mediating the killing of BCG under these conditions (Figure 6d).

### AECsup induces the production of reactive oxygen species (ROS)

**Figure 7:**
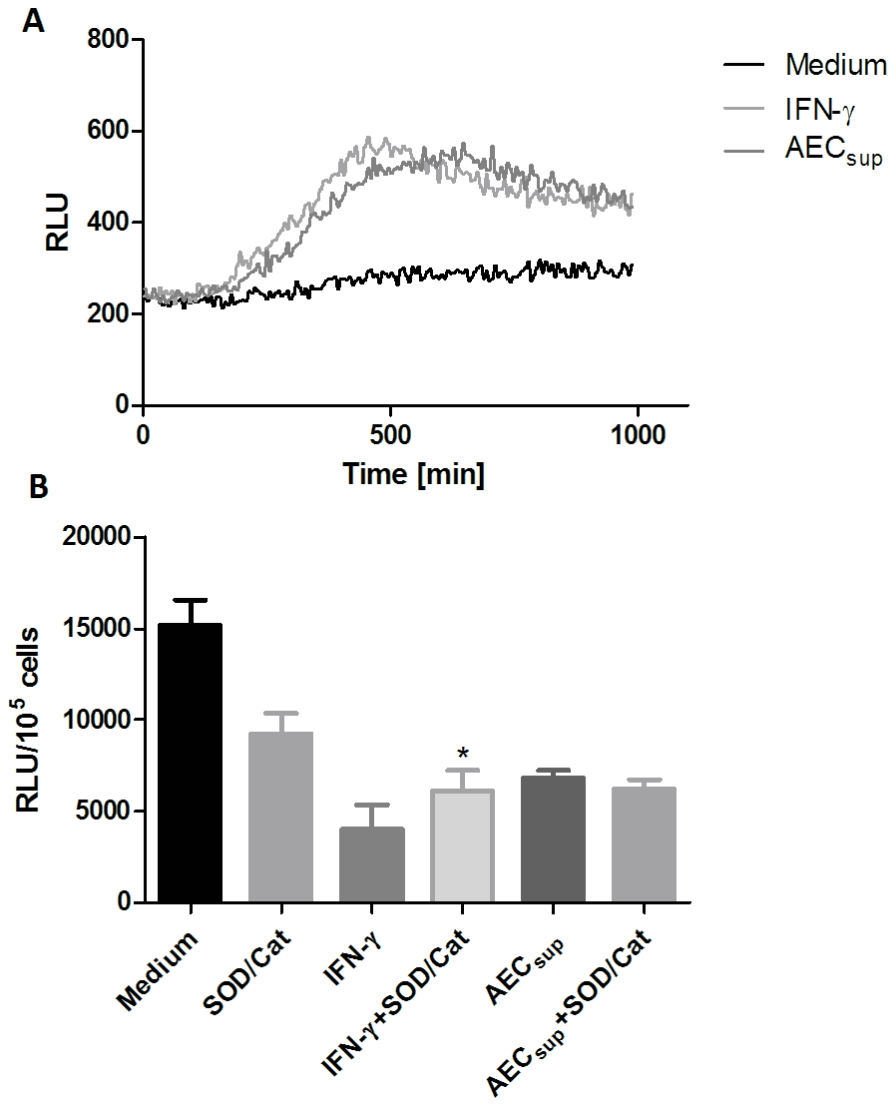
IFN-γ and AEC_sup_ induce an oxidative burst following infection with BCG. BMM were treated with IFN-γ (20 ng/ml) or AEC_sup_ 24 h prior to infection and then infected with GFP-BCG. a) ROS production was monitored in the cultures for 16 h by measuring luminescence with luminol as substrate. b) The effect of incubating cells with superoxide dismutase (SOD) and catalase on intracellular killing of BCG is shown. Values are expressed as means ± SD from 5 wells. Differences between treatments with or without SOD/catalase were analyzed using a paired t-test. * significant effect of SOD/catalase treatment, P<0.05.

Oxidative burst and the formation of ROS is another mechanism by which intracellular mycobacterial growth may be controlled [28]. Therefore, we next measured the production of ROS in our infection model. BMM treated with IFN-γ or AEC both displayed a significantly increased total production of ROS which peaked around 10 h after infection by BCG (Figure 7a). Using superoxide dismutase (SOD) and catalase; enzymes that convert superoxide to peroxide and peroxide to water and oxygen, respectively, did not affect the AEC_sup_-induced killing making it unlikely that the increased killing in cells incubated with AEC_sup_ was due to an increased oxidative burst (Figure 7b). On the other hand, treatment with SOD and catalase partially decreased the IFN-γ-induced killing perhaps because of its effect on the formation of peroxynitrites, as these have been shown to mediate killing of BCG [29].

### Choloroquine does not affect intracellular bacterial growth control promoted by AEC_sup_

**Figure 8:**
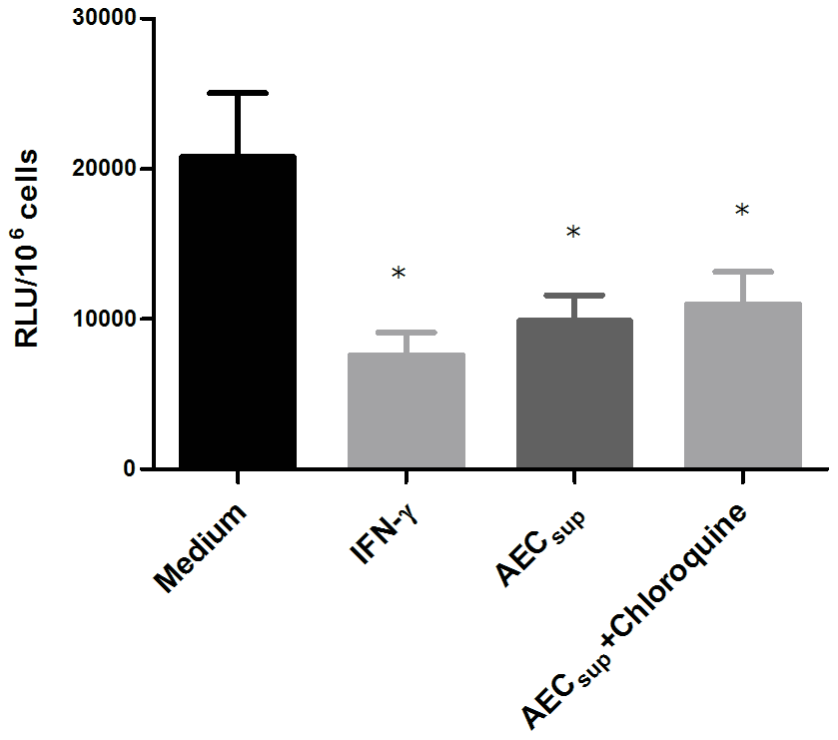
Inhibiting phagosomal acidification does not affect intracellular killing of BCG. BMM and PuM were infected with GFP-BCG. After infection, cells were treated with either IFN-γ or AEC_sup_ with or without chloroquine (10 µM). Bacterial growth was evaluated by determining RLU. Values are expressed as means ± SD from 5 wells. The data is representative of 2 independent experiments. Differences between treatments analyzed using a one-way ANOVA followed by Bonferroni's Multiple Comparison Test. * significantly different from medium control, P<0.05.

Since BCG reside in the phagosomal compartment we next addressed whether the increased intracellular killing might be because of increased processing through this compartment. For this, we treated BMM with chloroquine, an inhibitor of phagosomal acidification, after the infection but found that this treatment did not affect the intracellular bacterial control in macrophages receiving IFN-γ or AEC_sup_ (Figure 8). It is therefore unlikely that treatment with AEC_sup_ is acting by increasing phagosomal processing.

## Discussion

Macrophages control intracellular pathogens by various antimicrobial mechanisms such as phagosome acidification, induction of reactive oxygen and nitrogen species, phagosome fusion with lysosomes or by autophagy [27], [30], [31]. The ability of macrophages to control intracellular bacterial growth is fundamental in the defense of the respiratory tract therefore; we studied here the modulation of macrophage activity in the lung tissue. It is broadly accepted that IFN-γ is a potent activator of macrophages. We have also reported previously that factors secreted by AEC increase control of intracellular bacterial growth by macrophages [7]. In the current study, we aimed to investigate possible mechanisms responsible for this activation. The main findings shown are that; a) in contrast to other macrophage types, IFN-γ did not activate interstitial pulmonary macrophages to control intracellular bacterial growth even if the same macrophages could be activated by factors secreted by surrounding cells namely AEC; b) the unresponsiveness of PuM to IFN-γ was apparently regulated by SOCS1; c) the profile of cytokine secretion induced on macrophages by IFN-γ and AEC-derived factors was different and; d) in contrast to IFN-γ, intracellular bacterial destruction induced by AEC factors was not dependent on iNOS.

The Th1 cytokine IFN-γ, is able to induce downstream iNOS and consequently, a potent macrophage activator in the killing of intracellular mycobacteria [22].We first compared the microbicidal activity induced by IFN-γ on BMM and PuM infected with mycobacteria. BMM but not PuM were able to control intracellular growth of BCG after IFN-γ treatment. Pre-activation of PuM with IFN-γ before infection, did not revert the unresponsiveness of the PuM. Further, this was only observed in PuM and not in other cells of the respiratory tract such us alveolar macrophages or macrophages obtained from the peritoneal compartment. All this suggests that macrophages integrated in tissues are regulated differently than macrophages operating in secretions.

Interestingly, PuM were not completely unresponsive to IFN-γ since even to a lesser extent than for BMM, treatment with IFN-γ induced the secretion of NO and various cytokines such as IP-10, IL-12, IL-6 and TNF [32], [33] suggesting that PuM did express IFN-γ receptors but that transmission of signaling was ineffective as far as we could see only for the induction of intracellular killing. This is certainly noteworthy since PuM have the appropriate “killing machinery” successfully induced upon treatment with AEC-derived factors. Even if we do not have a formal explanation for this selective un-responsiveness of PuM to IFN-γ, it may be speculated that these cells can be more restricted in the activation by inflammatory mediators to prevent tissue damage in the lungs. Moreover, unresponsiveness of interstitial macrophages (PuM) to IFN-γ is intriguing and may open a new way of thinking in the current evaluation of the importance of IFN-γ in the defense against tuberculosis and other lung infections. PuM may play a more regulatory role limiting local inflammatory responses [34]-[37] in contrast to AM, which are described to behave as a classical macrophage taking up most of the particulate material that is delivered to the alveolar space [35], [38], [39].

We have reported before [7] that AEC-secreted factors induce expression of MMR and promote an elongated shape on macrophages, both aspects compatible with the phenotype of alternatively activated M2 macrophages and different from the M1 phenotype induced by IFN-γ. M2 macrophages are associated with Th2 immune responses and are considered to play more immune modulatory than microbicidal roles [40]-[43]. However, in contrast to this classic M2 phenotype, the macrophage type induced by AEC factors has also potent microbicidal properties suggesting a type of intermediate M1/M2 macrophage possibly required in tissue responses as has been proposed by others [44], [45]. Supporting this notion, an intermediate phenotype, M2b, was found to be important in the response and clearance of the intracellular pathogen *Leishmania infantum* [46]. In the same study, C-type lectins such as Dectin-1and MR were found to be crucial for the microbicidal response against this pathogen through ROS and caspase 1 induced IL-1β secretion pathways [46].

Although, IFN-γ is important in mediating macrophage activation, uncontrolled stimulation will not be beneficial to the host. Therefore, IFN-γ signaling is regulated by proteins such as SOCS1 identified as inducible feedback inhibitors of cytokines and consequently important for the regulation of inflammatory responses [25]. Thus, we evaluated whether SOCS1 proteins were involved in the un-responsiveness of PuM to IFN-γ by testing the response in IFN-γ^-/-^SOCS1^-/-^ mice. Interestingly, PuM from these mice responded to IFN-γ and efficiently controlled the intracellular bacteria. This showed not only that the transmission of IFN-γ signals was regulated by SOCS1 but again that PuM were capable of responding to IFN-γ.

Since iNOS and Arg-1 are co-regulated, we followed their induction on PuM and BMM after treatment with IFN-γ or AEC-derived media. AEC_sup_ induced Arg-1 on both cell-types but iNOS was only detected after treatment with IFN-γ. This lack of upregulation of iNOS expression, together with the lack of effect of NMMLA, a NOS inhibitor [47], all strongly indicate that mechanisms independent of the iNOS pathway were involved in the macrophage microbicidal activation by AEC factors. The profiles were also different regarding expression of SOCS1, only induced upon IFN-γ activation in both cell types. SOCS1 have been shown to be important in determining macrophage phenotype and function and regulate the balance between Arg-1 and iNOS activity. SOCS1 are also associated with the induction of Arg-1 in IL-4 treated macrophages [48]. Contrary to the IL-4-induced M2 macrophages described by Whyte et al. we show that AEC-derived factors induced Arg-1 in the absence of SOCS1 expression. Most important, AEC-derived factors promoted control of intracellular bacterial growth in PuM in a SOCS1 independent manner demonstrating that none of the factors secreted by AEC were involved in the induction of SOCS1 despite the fact that AEC-derived factors induced high expression of MMR and Arg-1 similar to M2-like macrophages [7].

To explore other possible mechanisms for the control of intracellular bacterial growth promoted by AEC factors, we looked at the formation of reactive oxygen species ROS and to IL-1β involved in autophagy related mechanisms. AEC factors were efficient inducers of IL-1β but, in contrast to what was reported by other groups [27], IL-1β was not responsible for the induced intracellular killing in our experimental setup. In BMM, the intracellular BCG growth was not affected by addition of exogenous IL-1β to the cultures. Moreover, BMM from caspase 1^-/-^ mice, unable to produce mature IL-1β, efficiently responded to stimulation by AEC factors.

ROS formation may also be important in the control of BCG infection through the oxidative burst [28]. To a similar extent as IFN-γ, AEC factors were efficient inducers of ROS but by themselves did not contribute to the control of intracellular BCG growth. Finally, we investigated a possible effect on the phagosomal compartment by treating BMM with chloroquine, an inhibitor of phagosomal acidification but we could not observe a major difference between the treated and the untreated groups.

We have been unable to directly ascribe the effect of AEC factors to the most common mechanisms operating separately. Thus, it may be that other mechanisms, not considered herein are responsible for these effects. An alternative explanation is that the combination of different microbicidal events and pathways could be operating in mycobacterial killing as described by Lefevre et al. where ROS contribute partially to IL-1β secretion in the responses to *L. infantum* [46].

Collectively, our data show that PuM are restricted in inflammatory responses mediated by IFN-γ through SOCS1 and that AEC-secreted factors enhance the microbicidal capacities of macrophages by iNOS-independent mechanisms that may be linked with to an intermediate M1-M2-like macrophage.

## Supporting Information

Figure S1 **Effect of either pretreating PuM with IFN-γ before (IFN-γ Pre Inf), or after infection (IFN-γ Post Inf).** PuM were either pretreated with IFN-γ (20 ng/ml) for 24 h before infection or left untreated. Cells were then infected with GFP-BCG for 4 h. After infection, cells were thoroughly washed and treated with gentamicin for 30 min. After additional washing, cells were either cultured in complete medium (Medium) or in medium with IFN-γ (20 ng/ml) or in medium with AEC-derived supernatant (AEC_sup_ 1∶2 diluted) for 48 h. Bacterial growth was evaluated by determining RLU in cell lysates. Data are shown as RLU/10^6^. Values are means ± SD of the mean from 2 independent experiments. Differences were analyzed with a one-way ANOVA. * significantly different from Medium.
(TIF)
