# Correction: Macrophage Control of Phagocytosed Mycobacteria Is Increased by Factors Secreted by Alveolar Epithelial Cells through Nitric Oxide Independent Mechanisms

**DOI:** 10.1371/journal.pone.0108198

**Published:** 2014-09-09

**Authors:** The PLOS ONE Staff

## Notice of Republication

This article was republished on August 25, 2014, to correct Figure 4, which was erroneously resized during the typesetting process. The publisher apologizes for the errors. Please view this article again to download the correct version. The originally published, uncorrected article and the republished, corrected article are provided here for reference.

## Supporting Information

File S1 Originally published, uncorrected article.
(PDF)

File S2 Republished, corrected article.
(PDF)

## References

[1] Petursdottir DH, Chuquimia OD, Freidl R, Fernández C (2014) Macrophage Control of Phagocytosed Mycobacteria Is Increased by Factors Secreted by Alveolar Epithelial Cells through Nitric Oxide Independent Mechanisms. PLoS ONE 9(8): e103411 doi:10.1371/journal.pone.0103411 25089618 10.1371/journal.pone.0103411 PMC4121081

