# Supplementary figures and images for: Macrophage Control of Phagocytosed Mycobacteria Is Increased by Factors Secreted by Alveolar Epithelial Cells through Nitric Oxide Independent Mechanisms
Source: PLoS One. 2014 Aug 4;9(8):e103411. doi: 10.1371/journal.pone.0103411 (PMC4121081; doi:10.1371/journal.pone.0103411)

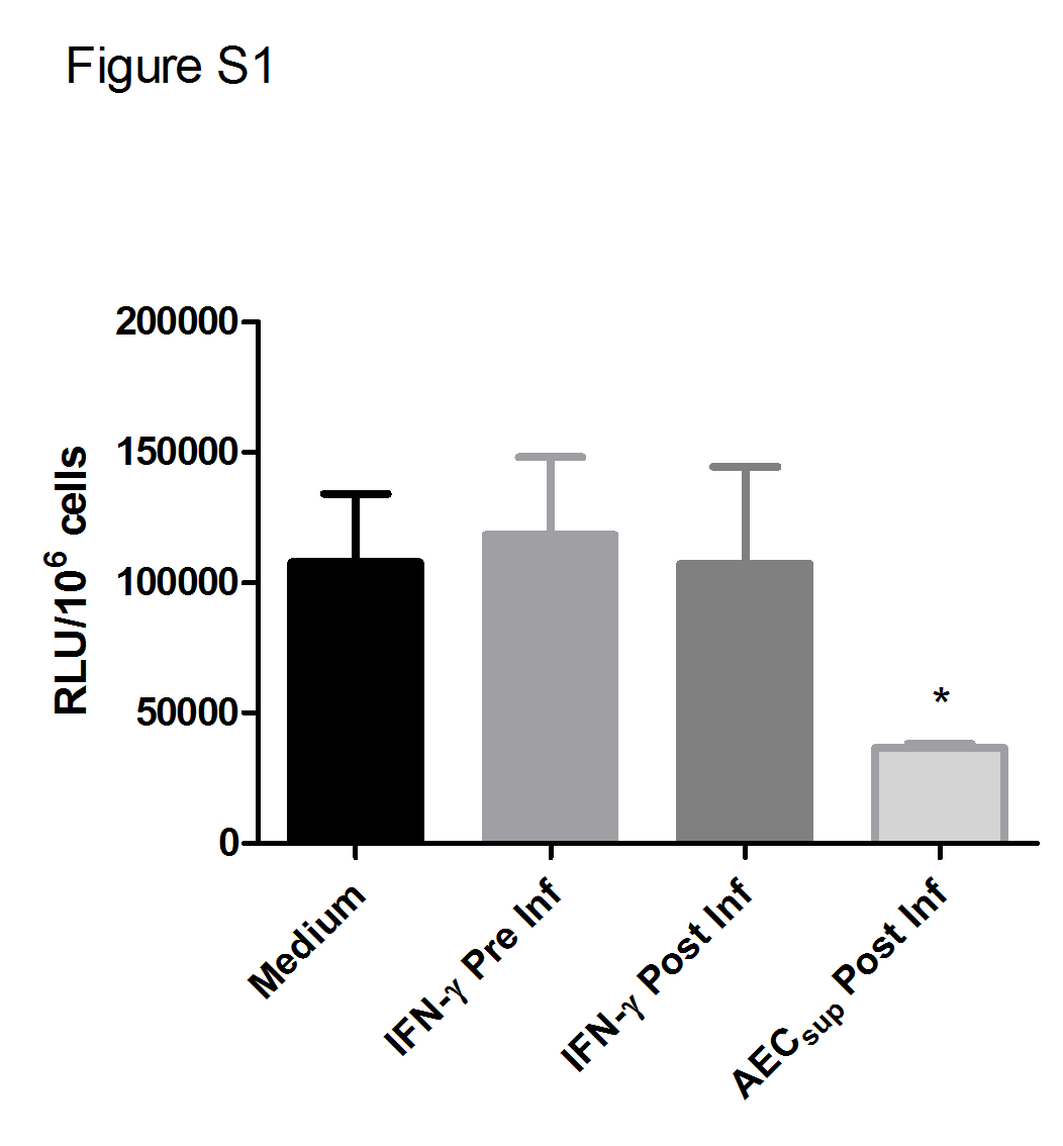

Supplement: Figure S1 — Effect of either pretreating PuM with IFN-γ before (IFN-γ Pre Inf), or after infection (IFN-γ Post Inf). PuM were either pretreated with IFN-γ (20 ng/ml) for 24 h before infection or left untreated. Cells were then infected with GFP-BCG for 4 h. After infection, cells were thoroughly washed and treated with gentamicin for 30 min. After additional washing, cells were either cultured in complete medium (Medium) or in medium with IFN-γ (20 ng/ml) or in medium with AEC-derived supernatant (AECsup 1∶2 diluted) for 48 h. Bacterial growth was evaluated by determining RLU in cell lysates. Data are shown as RLU/106. Values are means ± SD of the mean from 2 independent experiments. Differences were analyzed with a one-way ANOVA. * significantly different from Medium. (TIF) [file pone.0103411.s001.tif]
