# Supplementary material for: Correction: Macrophage Control of Phagocytosed Mycobacteria Is Increased by Factors Secreted by Alveolar Epithelial Cells through Nitric Oxide Independent Mechanisms
Source: PLoS One. 2014 Sep 9;9(9):e108198. doi: 10.1371/journal.pone.0108198 (PMC4159337; doi:10.1371/journal.pone.0108198)
Supplement: File S2 — Republished, corrected article. (PDF) [file pone.0108198.s002.pdf]

PLOS | ONE

Macrophage Control of Phagocytosed Mycobacteria Is Increased by Factors Secreted by Alveolar Epithelial Cells through Nitric Oxide Independent Mechanisms

Dagbjort H. Petursdottir*[¶], Olga D. Chuquimia[¶], Raphaela Freidl, Carmen Fernández

Department of Molecular Biosciences, The Wenner-Gren Institute, Stockholm University, Stockholm, Sweden

Abstract

Tissue-resident macrophages are heterogeneous with tissue-specific and niche-specific functions. Thus, simplified models of macrophage activation do not explain the extent of heterogeneity seen *in vivo*. We focus here on the respiratory tract and ask whether factors secreted by alveolar epithelial cells (AEC) can influence the functionality of resident pulmonary macrophages (PuM). We have previously reported that factors secreted by AEC increase control of intracellular growth of BCG in macrophages. In the current study, we also aimed to investigate possible mechanisms by which AEC-derived factors increase intracellular control of BCG in both primary murine interstitial macrophages, and bone marrow-derived macrophages and characterize further the effect of these factors on macrophage differentiation. We show that; a) in contrast to other macrophage types, IFN-γ did not increase intracellular growth control of *Mycobacterium bovis*, Bacillus Calmette-Guérin (BCG) by interstitial pulmonary macrophages although the same macrophages could be activated by factors secreted by AEC; b) the lack of response of pulmonary macrophages to IFN-γ was apparently regulated by suppressor of cytokine signaling (SOCS)1; c) AEC-derived factors did not induce pro-inflammatory pathways induced by IFN-γ e.g. expression of inducible nitric oxide synthase (iNOS), secretion of nitric oxide (NO), or IL-12, d) in contrast to IFN-γ, intracellular bacterial destruction induced by AEC-derived factors was not dependent on iNOS transcription and NO production. Collectively, our data show that PuM were restricted in inflammatory responses mediated by IFN-γ through SOCS1 and that factors secreted by AEC- enhanced the microbicidal capacities of macrophages by iNOS independent mechanisms.

Citation: Petursdottir DH, Chuquimia OD, Freidl R, Fernández C (2014) Macrophage Control of Phagocytosed Mycobacteria Is Increased by Factors Secreted by Alveolar Epithelial Cells through Nitric Oxide Independent Mechanisms. PLoS ONE 9(8): e103411. doi:10.1371/journal.pone.0103411

Editor: Joyoti Basu, Bose Institute, India

Received March 6, 2014; **Accepted** June 30, 2014; **Published** August 4, 2014

Copyright: © 2014 Petursdottir et al. This is an open-access article distributed under the terms of the Creative Commons Attribution License, which permits unrestricted use, distribution, and reproduction in any medium, provided the original author and source are credited.

Funding: This work was supported by grant FP7 (HEALTH-F3-2008-200732) from the European Commission. The funders had no role in study design, data collection and analysis, decision to publish, or preparation of the manuscript.

Competing Interests: The authors have declared that no competing interests exist.

* Email: dagbjort.petursdottir@su.se

¶ These authors contributed equally to this work.

Introduction

Macrophages are essential elements in both innate and adaptive immunity playing a crucial role in host defense. In addition to being a first line of resistance against pathogens, macrophages contribute to remodeling and repair. Tissue-resident macrophages are a heterogeneous population of immune cells that fulfill tissue-specific and niche-specific functions. These range from dedicated homeostatic functions, such as clearance of cellular debris and iron processing, to central roles in tissue immune surveillance, response to infection and the resolution of inflammation. Understanding the mechanisms that dictate tissue macrophage heterogeneity should explain why simplified models of macrophage activation do not explain the extent of heterogeneity seen *in vivo*.

In the lung tissue, although a number of immune cells secrete many of these factors, alveolar epithelial cells (AEC) and other non-immune cells are also able to produce a broad variety of factors including cytokines and chemokines that affect inflammatory responses in macrophages [1–6]. We have reported that factors secreted by AEC can influence both the phenotype and other characteristics of resident pulmonary macrophages (PuM) [7], confirming that the micro-environment provided by cytokines and other factors contribute to macrophage polarization and hence to their antimicrobial properties.

Polarized macrophages have been broadly classified into two groups: M1 (classically activated macrophages) or M2 (alternatively activated macrophages) [8]. M1 macrophages respond to type 1 inflammatory cytokines and microbial products such as lipopolysaccharide (LPS) to active inducible nitric oxide synthase (iNOS) and the production of reactive oxygen species (ROS) [8–10]. Thus, M1 cells are thought to represent a "common host response" for microbicidal capacities [8–10] while M2 cells are immunoregulatory with poor microbicidal activity [8]. Since M2 macrophages display a very broad functional spectrum, a further subcategorization has been proposed based on the *in vitro* stimuli used for the generation of the different phenotypes, where culturing with IL-4 or IL-13 yields M2a; induced by immune complexes, TLR agonists or IL-1R yield M2b; IL-10 or glucocorticoids yield M2c [11].

Previously, we have shown that treatment of PuM with AEC-derived factors affect their functional properties seen e.g. in morphological alterations demonstrated by elongated cell shape as well as increased expression of the marker macrophage mannose

receptor (MMR), both features indicating M2 polarization [7,12,13]. However, treatment with AEC-derived factors also increases the PuM phagocytic activity as well as their capacity to control mycobacteria intracellular growth upon infection with *Mycobacterium bovis*, Bacillus Calmette-Guérin (BCG), characteristics more related to M1 polarization [7].

In the present study we investigated possible mechanisms for the induction of intracellular bacterial growth control in macrophages mediated by AEC-derived factors and compared this with treatment with IFN-γ, a classical inducer of M1 activation. Our data suggested that AEC-derived factors induced microbicidal functions through iNOS-independent mechanisms and that suppressor of cytokine signaling 1 (SOCS1), a known inhibitor of IFN-γ signaling, [14]was involved in the inability of PuM to respond to IFN-γ.

Materials and Methods

Mice

All mouse experiments were approved and performed in accordance with the guidelines of the Animal Research Ethics Board at Stockholm University (ethical approval ID: N463/12). The studies were performed using 8–12-week-old female C57BL/6 mice purchased from NOVA-SCB, Sweden. All animals were kept at the Animal Department of the Arrhenius Laboratories, Stockholm University, Sweden and housed in pathogen-free conditions. Mice were supervised daily and sentinel mice were used to assess and ensure pathogen free conditions in the facility. IFN-$\gamma^{-/-}$SOCS1$^{-/-}$ mice were kindly provided by M. Rottenberg (MTC, Karolinska Institutet, Stockholm, Sweden) and experiments conducted in accordance with the guidelines of Karolinska Institutet.

Bacteria

We used BCG, transformed with a dual reporter plasmid containing the human codon-optimised and fluorescence-enhanced EGFP and the bacterial luciferase A and B (luxAB) genes from *Vibrio harveyi* [15] herein called GFP-BCG. Bacterial contents are expressed as relative luminescence units (RLU). The increase of OD and RLU was followed for all batches and experiments. All batches were also routinely controlled for CFU and RLU values. The correlation between CFU and RLU has been previously published as supplementary data [16,17]. Furthermore, a correlation between CFU and RLU has even been confirmed in the experimental setup used in this study, i.e. in an assay determining intracellular survival of bacteria within macrophages (unpublished data). GFP-BCG was grown in Middlebrook 7H9 broth (DIFCO, Sparks, MD, USA) supplemented with albumin-dextrose-catalase (ADC), 0.5% glycerol, 0.05% Tween 80 (vol/vol), and 50 µg/ml hygromycin for 10–15 days. Bacteria were collected at a log phase of growth (absorbance 1.0 measured at OD_{650}) and frozen in PBS with 10% glycerol and kept at −80°C. Before infection of cell cultures, a vial was thawed and placed in culture as above for 4–5 days, reaching an early log phase (OD_{650} ~0.3) corresponding to approximately 3×10^8 CFU per ml. To determine the RLU, Decanal (0.01% v/v in ethanol) (Sigma-Aldrich) was used as a specific substrate for the bacterial enzyme luxAB. The samples were mixed immediately and luminescence measured after 15 sec in a Modulus, Turner Bio Systems luminometer.

Isolation of alveolar epithelial cells (AEC) and interstitial pulmonary macrophages (PuM)

Total pulmonary cells were prepared using Corti's protocol [18] with previously described modifications [16,17]. PuM were isolated from either C57BL/6 or IFN-$\gamma^{-/-}$SOCS1$^{-/-}$ mice [19] by positively selecting CD45$^+$ cells from total pulmonary cells using MACS (Miltenyi Biotec, Bergish Gladbach, Germany) and a subsequent adhesion isolation. After isolation, cells were cultured for 48 h in RPMI (Gibco-Invitrogen, Paisley, UK) supplemented with 10% FCS, 2 mM L-glutamine, 100 U/ml penicillin, 100 µg/ml streptomycin, 0.02 M Hepes, and 0.05 M 2-mercaptoethanol (Sigma) (complete RPMI) at 37°C and 5% CO_2. Next, cells were washed to remove non-adherent cells and debris. In average, 98% of the cells were positive for the macrophage marker F4/80, as determined by flow cytometry. AEC were obtained by depleting CD45$^+$ and CD146$^+$ cells from lung cell preparations using MACS. In average, 92-95% of these CD45$^-$CD146$^-$ cells exhibited an AEC phenotype, where approximately 22% expressed podoplanin (type I AEC) and approximately 72% expressed CD74 (type II AEC) as determined by flow cytometry. Cell viability was determined by trypan blue exclusion to be over 90%.

For the preparation of AEC-derived media, AEC were plated (5×10^4 cells/well) in 96-well flat bottom plates (Costar, NY, USA) and cultured for 48 h. After washing the cells three times, cells were again kept unstimulated for 24 h. These cell culture supernatants were collected, pooled, filtered through a 0.2 µm filter and stored at −70°C until use. These cell culture supernatants were named AEC_{sup}.

Isolation of alveolar macrophages (AM) and peritoneal exudate macrophages (PEM)

AM were isolated from bronchoalveolar lavage (BAL) using a previously described protocol [20] with some modifications. Briefly, a bronchoalveolar lavage (BAL) was performed with 1–2 ml of pre-warmed (37°C) PBS-0.5 mM EDTA (Gibco) via the trachea 3-4 times. After lavage, BAL fluids were pooled and put on ice. PEM were isolated as previously described [20]. The mouse's skin was removed carefully leaving the peritoneal membrane intact. The peritoneal cavity was rinsed twice with RPMI (5 ml) and the collected peritoneal exudate cells were pooled and placed on ice. BAL and peritoneal exudate cells (PEC) were then washed twice in ice-cold RPMI and plated in 48-well flat bottom plates (Costar, NY, USA) at a cell concentration of 2×10^5 cells/well (BAL) and 1.5×10^6 cells/well (PEC) for 2 h in complete RPMI. Cells were washed 2–3 times to eliminate debris and non-adherent cells and cultured in complete RPMI overnight. After 24 h, ~98 ± 1% of BAL cells and 98 ± 1% of PEC were positive for the macrophage marker F4/80 and were considered as AM and PEM, respectively. A further characterization showed that, 83 ± 8% of AM were F4/80$^+$ CD11c$^+$, a typical AM phenotype, 15 ± 3% were F4/80$^+$CD11b$^+$, and 8±1% were F4/80$^+$CD11b$^+$CD11c$^+$ whereas approximately 99 ± 1% of PEM were F4/80$^+$ CD11b$^+$.

Generation of bone marrow derived macrophages (BMM)

BMM were harvested and frozen as previously described [21]. After thawing, the bone marrow cells were cultured in complete RPMI supplemented with 20% L929 cell conditioned medium (as a source of macrophage colony-stimulating factor) and cultured for 6–7 days, replacing medium every second day. Before use, growth factor-containing media were removed and the BMM cultured in complete RPMI for 24 h. BMM from caspase 1$^{-/-}$ mice were kindly provided by S. Musciol and B. Henriques-Normark (MTC, Karolinska Institutet, Solna, Sweden)

Infection and intracellular bacterial growth

BMM, PuM, AM and PEM were cultured in 48 or 12-well plates as described above 1×10^5 and 4×10^5 cells per well respectively. Cells were kept with complete RPMI without antibiotics for 24 h prior to infection with GFP-BCG at a MOI 10:1 (bacteria:cell) for 4 h. After infection, cells were washed three times and treated with gentamicin (100 µg/ml) for 30–60 min at 37°C. After further washing, cells were either kept untreated or treated with 20 ng/ml of IFN-γ (Mabtech, Sweden), or IL-1β (1 or 10 ng/ml) or AEC_{sup} (diluted 1:2 in cell culture media). For inhibition of iNOS 1 mM of N^G-monomethyl-L-arginine (NMMLA) (Sigma) was added together with either IFN-γ or AEC_{sup}. For reducing reactive oxygen species (ROS) in the cultures, cells were incubated with the ROS scavengers superoxide dismutase (SOD, 15 U/ml, Sigma-Aldrich) and catalase 500 µg/ml (Sigma-Aldrich). The bacterial loads were measured by determining RLU and data is displayed as RLU/10^6 cells.

Real- time quantitative PCR

Total RNA was harvested from BMM or PuM untreated or treated with either IFN-γ or AEC_{sup} at 4 or 24 h after the end of BCG infection using QIAzol Lysis Reagent (Qiagen). After isolation, the RNA was run on a 1% agarose gel to verify that the RNA was intact. RT-PCR was performed on 1 µg of RNA using SuperScript III Reverse Transcriptase (Invitrogen) with Oligo(dT)$_{20}$ Primers (Invitrogen). Transcripts were quantified on a RotorGene 6000 real-time PCR cycler (Corbett Life Sciences) using Kapa Sybrfast qPCR Kit (Kapa Biosystems). Primer sequences used were: iNOS sense: CAGCTCGGCTGTACAAACCTT, antisense: CATTGGAAGTGAAGCGTTTCG, IL-1β sense: TGGTGTGTGACGTTCCCATT antisense: CAGCACGAGGCTTTTTTGTTG, Arg-1 sense: GCAGTGGCTTTAACCTTGGC, antisense: TGGCGCATTCACAGTCACTT. HPRT sense CCC AGC GTC GTG ATT AGC, antisense GGA ATA AAC ACT TTT TCC AAA TCC was used as a control gene and used to calculate dCt values. To determine the effect of treatment with IFN-γ or AEC_{sup}, values were normalized to that of cells infected with BCG receiving no treatment and the fold induction (RQ) calculated.

Cytokine and nitric oxide (NO) determinations

Cytokines and nitrite (an indirect indicator of NO production) were measured in cell culture supernatants from BMM and PuM un-treated or treated with either IFN-γ or AEC_{sup} at 4, 24 and 48 h after the end of infection with BCG. For ELISA determinations, the commercially, TNF, IL-6, CXCL10/Interferon gamma-induced protein 10 kDa (IP-10), IL-1β (R&D Systems) and IL-12, (Mabtech) were used to determine the cytokine levels in the culture supernatants according to the manufacturer's recommendations. The enzyme-substrate reaction was developed using p-nitrophenyl phosphate (Sigma) for IL-12 and tetramethylbenzidine substrate (R&D Systems) for the rest of determinations. Depending on the substrate used, the optical density was measured in a multiscan ELISA reader (Anthos Labtech Instruments, Salzburg, Austria) at 405 or 450 nm. NO production was determined by measuring nitrite concentration using the Griess reaction according to the manufacturers protocol (Sigma).

Analysis of ROS production

BMM were prepared as described above and then pretreated with either IFN-γ (20 ng/ml) or AEC_{sup} (diluted 1:2 in cell culture medium) for 24 h. Cells were then infected with BCG (MOI 1:10) and 500 µM luminol (Sigma) added to the cultures. Plates were then placed in an EnSpire Multimode Plate Reader (PerkinElmer) and luminescence monitored at 37°C every 3 min for 15 h.

Statistical analysis

Data are presented as the mean ± SD. Differences between treatments in the groups were analyzed using one-way ANOVA followed by Bonferroni's post-test for multiple comparisons. For qPCR data differences between treatment at different time-points were analyzed using a non-parametric one-way ANOVA (Kruskal-Wallis) with Dunn's post-test. Differences were considered significant at $p<0.05$. All data were analyzed using the GraphPad InStat version 5.0 (GraphPad Software, San Diego, CA, USA).

Results

Impaired control of intracellular BCG growth by PuM upon IFN-γ treatment

Since IFN-γ is known to mediate the microbicidal activation of macrophages against mycobacteria [22] we evaluated the response of PuM and BMM to IFN-γ. BMM and PuM were infected with GFP-BCG as described in Materials and Methods, treated with different concentrations of IFN-γ (20, 40 and 80 ng/ml) or left untreated. Bacterial loads were monitored by measuring RLU. As expected, BMM reduced the intracellular bacterial content upon treatment with IFN-γ (Figure 1). In contrast, PuM were not able to reduce bacterial load even after treatment with doses of IFN-γ as high as 80 ng/ml (Figure 1). We further evaluated whether PuM needed to be activated prior to mycobacterial infection since it has been described that IFN-γ "activated" macrophages could better control mycobacteria growth [23,24]. For this, PuM were treated with IFN-γ prior infection and bacterial loads monitored. We found that PuM pre-treated with IFN-γ were still unable to control intracellular BCG growth (Fig S1).

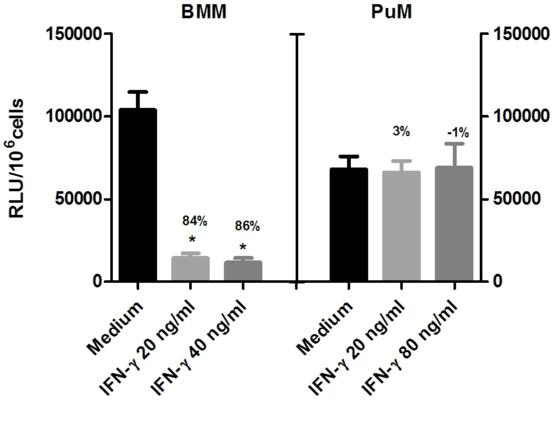

Figure 1. Impaired intracellular control of BCG growth by PuM upon IFN-γ treatment. BMM and PuM were infected with GFP-BCG. After infection, cells were left untreated or treated with IFN-γ (20, 40 and 80 ng/ml) for 48 h. Bacterial growth was evaluated by determining RLU. Data are shown as % reduction of phagocytosed bacteria evaluated as RLU. Values are means ± SD of the mean of a representative experiment from 3 independent experiments with 4 replicates each. The differences between groups of BMM and PuM were analyzed using a one-way ANOVA followed by Bonferroni's Multiple Comparison Test, * significantly different from medium control, P<0.05.
doi:10.1371/journal.pone.0103411.g001

PuM are specifically unresponsive to IFN-γ in the control of intracellular bacterial growth

To understand whether the unresponsiveness to IFN-γ was a characteristic of pulmonary macrophages, we compared PuM with other tissue derived macrophages, namely AM and PEM obtained from lung and peritoneal exudates, respectively. Macrophages were treated with IFN-γ and with AEC-derived factors (AEC_{sup}) since we have described previously [7] that treatment with AEC-derived media decreased intracellular bacterial loads in PuM. AM, PuM and PEM were isolated and infected with GFP-BCG and later, either left untreated or treated with IFN-γ or AEC_{sup}. Bacterial loads were monitored by measuring RLU. In contrast to the effect on PuM, IFN-γ was able to induce a significant control of intracellular BCG growth in AM and PEM (49% and 45% reduction respectively) (Figure 2). Importantly, treatment with AEC_{sup} mediated reduction of intracellular BCG in the three types of primary macrophages, PuM, AM and PEM (Figure 2) indicating that PuM was specifically unable to respond to IFN-γ.

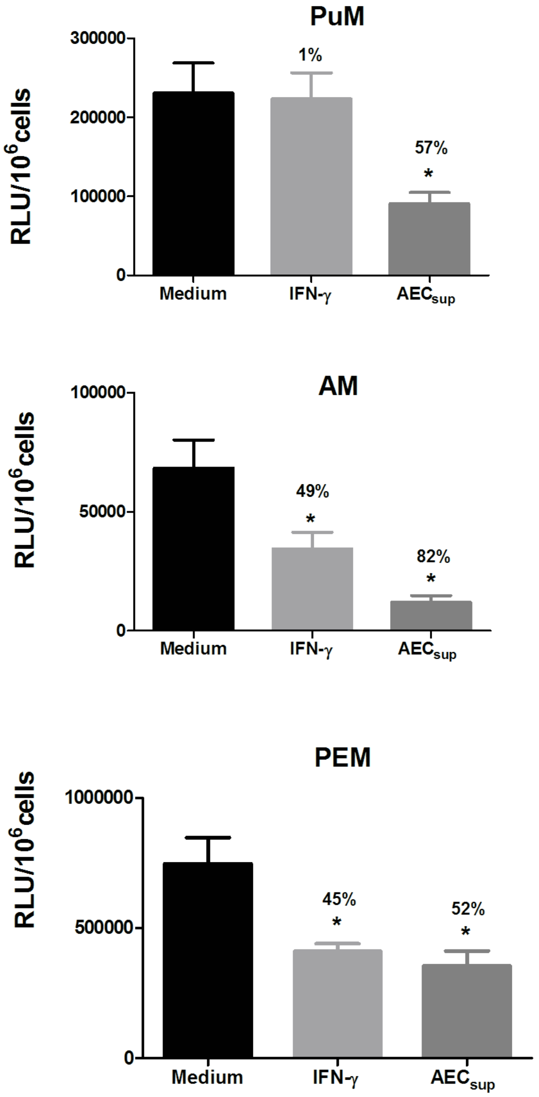

Figure 2. PuM are specifically unresponsive to IFN-γ in the control of intracellular bacteria. PuM, AM and PEM were infected with GFP-BCG. After infection, cells were treated with either IFN-γ or AEC_{sup} for 48 h or left untreated. Bacterial growth was evaluated by determining RLU. Data are shown as % reduction of phagocytosed bacteria evaluated as RLU. Values are means ± SD of the mean of 2 independent experiments with 4 replicates each. The differences between groups of PuM, AM and PEM were analyzed using a one-way ANOVA followed by Bonferroni's Multiple Comparison Test, * significantly different from medium control, $P<0.05$.
doi:10.1371/journal.pone.0103411.g002

IFN-γ and AEC_{sup} induce different cytokine profiles in PuM and BMM

To further understand the different responses elicited in PuM and BMM by AEC_{sup} and IFN-γ, we stimulated the two cell types *in vitro* as described in Materials and Methods and collected supernatants 4 and 24 h later. We monitored the production of IL-12, IP-10 and IL-6 since these factors are associated with IFN-γ mediated signaling through the activation of receptor-associated JAKs. Two major observations are obvious from the data shown in Figure 3. Firstly, comparing the stimulation capacity of IFN-γ and AEC_{sup}, the secretory profile was different in both cell types. AEC_{sup} did not induce production of IL-12 and IP-10 statistically above that of unstimulated cells but induced IL-6 secretion significantly in BMM at 4 h. Similarly, AEC_{sup} also tended to increase IL-6 secretion in PuMSecondly, although the profile of stimulation was similar, the secretion of all factors measured was lower in PuM compared with BMM.

Impaired intracellular control of BCG by PuM upon IFN-γ treatment is regulated by SOCS1

To assess whether the reduced capacity of PuM to respond to IFN-γ was related to intracellular regulations of signaling, we tested this in PuM derived from IFN-$\gamma^{-/-}$SOCS1$^{-/-}$ mice. SOCS1 has been described to be a critical inhibitor of IFN-γ signaling [25] and also able to dampen early responses to BCG and *Mycobacterium tuberculosis* [19]. Thus, we evaluated the effects of IFN-γ and AEC_{sup} treatments on intracellular BCG control by PuM from IFN-$\gamma^{-/-}$SOCS1$^{-/-}$ mice. After IFN-γ treatment, PuM from IFN-$\gamma^{-/-}$SOCS1$^{-/-}$ mice controlled intracellular growth of BCG, to a similar extent as cells treated with $AEC_{sup,}$ indicating that the lack of response to IFN-γ is likely not due to lack of surface IFN-γ receptor expression but rather that the response is under regulation of SOCS1 (Figure 4a). However, there were no differences seen in SOCS1 expression between BMM and PuM and both cell types upregulated SOCS1 upon stimulation with IFN-γ indicating that the selective lack of response to IFN-γ in PuM was not due to a lack of SOCS1 but rather an event downstream of SOCS1 (data not shown). Treating cells with both IFN-γ and AEC_{sup} tended to show an additive effect suggesting independent mechanisms of intracellular bacterial control (Figure 4a). Similarly to the results observed in the wild type mice, upon treatment with IFN-γ, the levels of IL-12 induced in PuM derived from IFN-$\gamma^{-/-}$SOCS1$^{-/-}$ mice were lower than in BMM (data not shown) Thus, reduced IL-12 secretion may not only be subjected to SOCS1 regulation but also to other mechanisms inherent to different tissue-derived macrophages.

AEC_{sup} increases intracellular growth control without an effect on iNOS

Up-regulation of iNOS and the subsequent synthesis of reactive nitrogen species is currently believed to be one of the primary mechanisms downstream of IFN-γ-induced killing of mycobacteria [22]. On the other hand, Arg-1 can compete with iNOS for their common substrate L-arginine, and consequently, the expression of iNOS and Arg-1 is often reciprocally regulated in macrophages [26]. To reveal possible mechanisms for the observed intracellular bacterial control, we followed the expression of iNOS and Arg-1

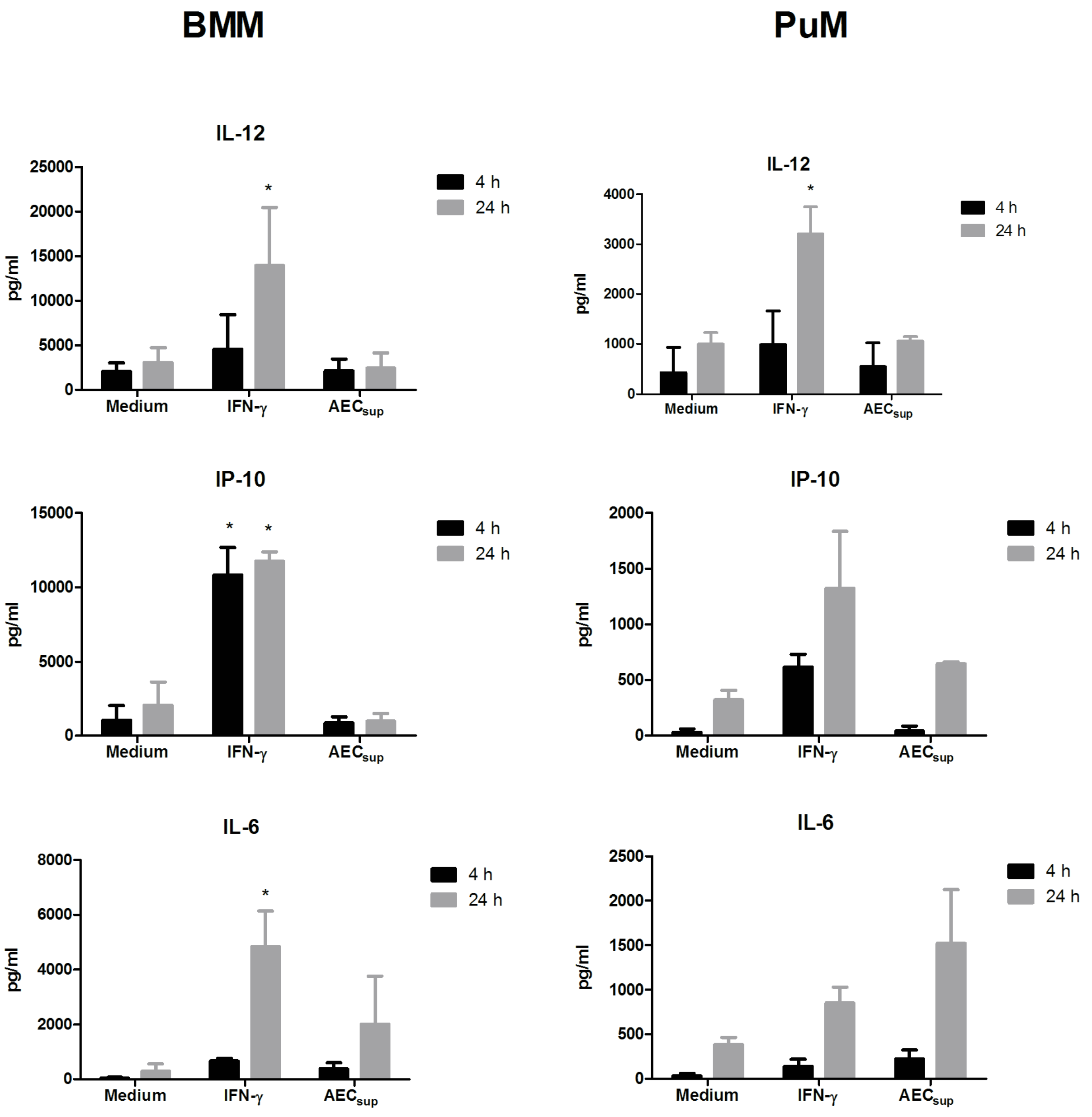

Figure 3. IFN-γ and AECsup induce different cytokine profiles in PuM and BMM. BMM and PuM were infected with GFP-BCG. After infection, cells were left untreated or treated with either IFN-γ or AEC_{sup}. Cell culture supernatants were collected at 4 and 24 h after treatments. IL-12, IP-10 and IL-6, levels were measured using ELISA. Values are expressed as means ± SD, from 3 independent experiments. The differences between groups of PuM and BMM were analyzed using a one-way ANOVA followed by Bonferroni's Multiple Comparison Test, * Significantly different from medium control, $p<0.05$.
doi:10.1371/journal.pone.0103411.g003

induced in PuM and BMM upon treatment with AEC_{sup} or IFN-γ. We found that IFN-γ but not AEC_{sup} induced iNOS in both BMM and PuM (Figure 5a). In contrast, in both PuM and BMM, Arg-1 was induced only by AEC_{sup} and not by IFN-γ (Figure 5b). Notably, the levels of Arg-1 induced in BMM were higher than the levels induced in PuM. Addition of NMMLA, an inhibitor of iNOS, to cultures of BMM infected with BCG and treated with AEC_{sup} or IFN-γ, inhibited the bacterial load reduction mediated by IFN-γ but had no effect on AEC_{sup} activity (Figure 5c) confirming that AEC_{sup} increased intracellular killing using an iNOS independent pathway.

AEC_{sup} induces IL-1β transcription and secretion

IL-1β has been shown to be involved in the intracellular killing of mycobacteria through the induction of autophagy related mechanisms [27]. We therefore investigated whether AEC_{sup} affect IL-1β production. We observed a difference in the profile of induction of this interleukin by the two stimuli where only AEC_{sup} induced IL-1β transcription (Figure 6a) and secretion (Figure 6b) above that of cells only infected with BCG. We next tested whether this increase in IL-1β secretion in AEC_{sup}-treated BMM might mediate the increased intracellular killing of BCG by these cells. However, addition of exogenous IL-1β to the cultures did not affect intracellular bacterial killing (Figure 6c). Finally, we asked

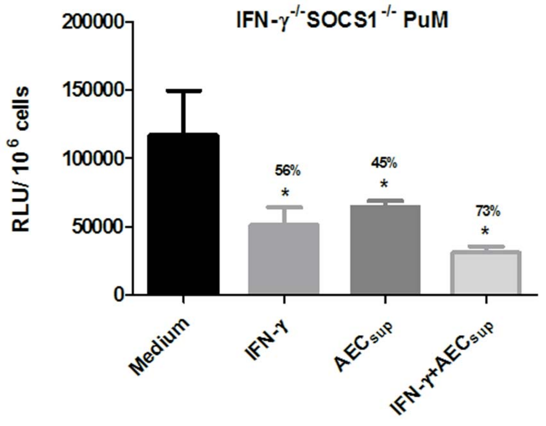

Figure 4. Impaired intracellular control of BCG growth by PuM upon IFN-γ treatment is regulated by SOCS1. PuM from IFN-$\gamma^{-/-}$ SOCS1$^{-/-}$ mice were infected with GFP-BCG. After infection, cells were left untreated or treated with IFN-γ, AEC_{sup} and IFN-γ + AEC_{sup} for 48 h. Bacterial growth was evaluated by determining RLU. Data are shown as % reduction of phagocytosed bacteria evaluated as RLU. Values are means ± SD of the mean of a representative experiment from 2 independent experiments with 4 replicates. The differences between groups of IFN-$\gamma^{-/-}$ SOCS1$^{-/-}$ PuM were analyzed using a one-way ANOVA followed by Bonferroni's Multiple Comparison Test. * significantly different from medium control, $P<0.05$.
doi:10.1371/journal.pone.0103411.g004

the same question using BMM derived from caspase 1$^{-/-}$ mice that are unable to secrete the mature form of IL-1β. We observed that AEC_{sup} activated BMM derived from caspase 1$^{-/-}$ mice were able to control efficiently intracellular bacteria growth supporting the notion that IL-1β was not the key molecule in mediating the killing of BCG under these conditions (Figure 6d).

AECsup induces the production of reactive oxygen species (ROS)

Oxidative burst and the formation of ROS is another mechanism by which intracellular mycobacterial growth may be controlled [28]. Therefore, we next measured the production of ROS in our infection model. BMM treated with IFN-γ or AEC both displayed a significantly increased total production of ROS which peaked around 10 h after infection by BCG (Figure 7a). Using superoxide dismutase (SOD) and catalase; enzymes that convert superoxide to peroxide and peroxide to water and oxygen, respectively, did not affect the AEC_{sup}-induced killing making it unlikely that the increased killing in cells incubated with AEC_{sup} was due to an increased oxidative burst (Figure 7b). On the other hand, treatment with SOD and catalase partially decreased the IFN-γ-induced killing perhaps because of its effect on the formation of peroxynitrites, as these have been shown to mediate killing of BCG [29].

Choloroquine does not affect intracellular bacterial growth control promoted by AEC_{sup}

Since BCG reside in the phagosomal compartment we next addressed whether the increased intracellular killing might be because of increased processing through this compartment. For this, we treated BMM with chloroquine, an inhibitor of phagosomal acidification, after the infection but found that this treatment did not affect the intracellular bacterial control in macrophages receiving IFN-γ or AEC_{sup} (Figure 8). It is therefore unlikely that treatment with AEC_{sup} is acting by increasing phagosomal processing.

Discussion

Macrophages control intracellular pathogens by various antimicrobial mechanisms such as phagosome acidification, induction of reactive oxygen and nitrogen species, phagosome fusion with lysosomes or by autophagy [27,30,31]. The ability of macrophages to control intracellular bacterial growth is fundamental in the defense of the respiratory tract therefore; we studied here the modulation of macrophage activity in the lung tissue. It is broadly accepted that IFN-γ is a potent activator of macrophages. We have also reported previously that factors secreted by AEC increase control of intracellular bacterial growth by macrophages [7]. In the current study, we aimed to investigate possible mechanisms responsible for this activation. The main findings shown are that; a) in contrast to other macrophage types, IFN-γ did not activate interstitial pulmonary macrophages to control intracellular bacterial growth even if the same macrophages could be activated by factors secreted by surrounding cells namely AEC; b) the unresponsiveness of PuM to IFN-γ was apparently regulated by SOCS1; c) the profile of cytokine secretion induced on macrophages by IFN-γ and AEC-derived factors was different and; d) in contrast to IFN-γ, intracellular bacterial destruction induced by AEC factors was not dependent on iNOS.

The Th1 cytokine IFN-γ, is able to induce downstream iNOS and consequently, a potent macrophage activator in the killing of intracellular mycobacteria [22].We first compared the microbicidal activity induced by IFN-γ on BMM and PuM infected with mycobacteria. BMM but not PuM were able to control intracellular growth of BCG after IFN-γ treatment. Pre-activation of PuM with IFN-γ before infection, did not revert the unresponsiveness of the PuM. Further, this was only observed in PuM and not in other cells of the respiratory tract such us alveolar macrophages or macrophages obtained from the peritoneal compartment. All this suggests that macrophages integrated in tissues are regulated differently than macrophages operating in secretions.

Interestingly, PuM were not completely unresponsive to IFN-γ since even to a lesser extent than for BMM, treatment with IFN-γ induced the secretion of NO and various cytokines such as IP-10, IL-12, IL-6 and TNF [32,33] suggesting that PuM did express IFN-γ receptors but that transmission of signaling was ineffective as far as we could see only for the induction of intracellular killing. This is certainly noteworthy since PuM have the appropriate "killing machinery" successfully induced upon treatment with AEC-derived factors. Even if we do not have a formal explanation for this selective un-responsiveness of PuM to IFN-γ, it may be speculated that these cells can be more restricted in the activation by inflammatory mediators to prevent tissue damage in the lungs. Moreover, unresponsiveness of interstitial macrophages (PuM) to IFN-γ is intriguing and may open a new way of thinking in the current evaluation of the importance of IFN-γ in the defense against tuberculosis and other lung infections. PuM may play a more regulatory role limiting local inflammatory responses [34-37] in contrast to AM, which are described to behave as a classical macrophage taking up most of the particulate material that is delivered to the alveolar space [35,38,39].

We have reported before [7] that AEC-secreted factors induce expression of MMR and promote an elongated shape on macrophages, both aspects compatible with the phenotype of alternatively activated M2 macrophages and different from the M1 phenotype induced by IFN-γ. M2 macrophages are associated

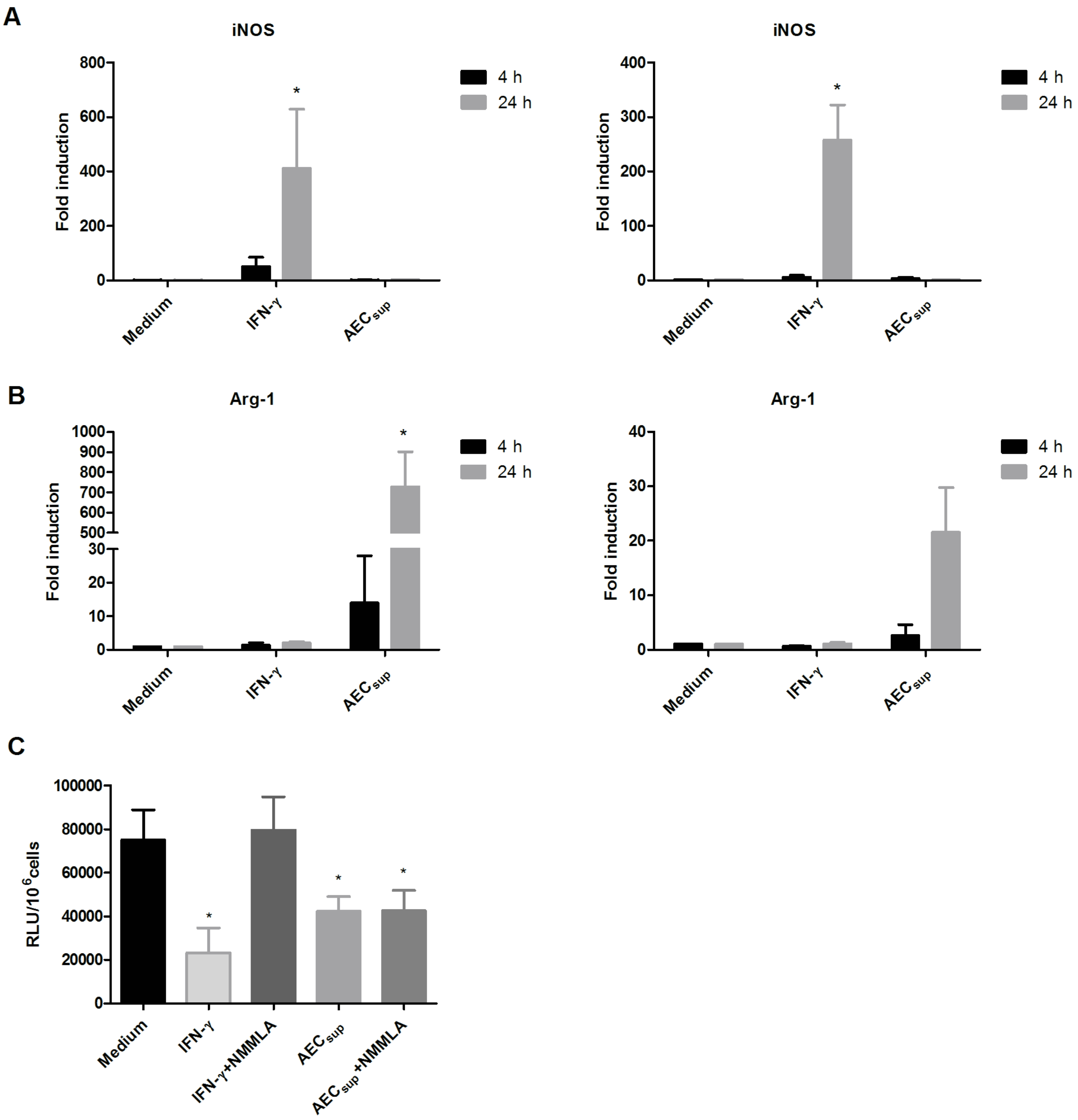

Figure 5. AEC_{sup} has opposite effects to IFN-γ on iNOS, and Arg-1 expression in BMM and PuM and mediates killing through an iNOS independent mechanism. BMM and PuM were infected with GFP-BCG. After infection, cells were treated with either IFN-γ or AEC_{sup} or 1 mM of NG-monomethyl-L-arginine (NMMLA) together with either IFN-γ or AEC_{sup} or left untreated. Total RNA was extracted from both cell-types after 4 and 24 h of treatment and the mean-fold accumulation of a) iNOS, b) Arg-1 ± SD from 3-4 experiments. In c) the effect of the iNOS inhibitor NMMLA on intracellular growth of BCG is shown. Bacterial growth was evaluated by determining RLU. Data are expressed as mean ± SD. The differences between groups of BMM and PuM were analyzed using non-parametric, one-way ANOVA (Kruskal-Wallis) with Dunn's post-test. * significantly different from medium control, $P<0.05$
doi:10.1371/journal.pone.0103411.g005

with Th2 immune responses and are considered to play more immune modulatory than microbicidal roles [40-43]. However, in contrast to this classic M2 phenotype, the macrophage type induced by AEC factors has also potent microbicidal properties suggesting a type of intermediate M1/M2 macrophage possibly required in tissue responses as has been proposed by others [44,45]. Supporting this notion, an intermediate phenotype, M2b, was found to be important in the response and clearance of the intracellular pathogen *Leishmania infantum* [46]. In the same study, C-type lectins such as Dectin-1and MR were found to be crucial for the microbicidal response against this pathogen through ROS and caspase 1 induced IL-1β secretion pathways [46].

Although, IFN-γ is important in mediating macrophage activation, uncontrolled stimulation will not be beneficial to the host. Therefore, IFN-γ signaling is regulated by proteins such as SOCS1 identified as inducible feedback inhibitors of cytokines and consequently important for the regulation of inflammatory responses [25]. Thus, we evaluated whether SOCS1 proteins

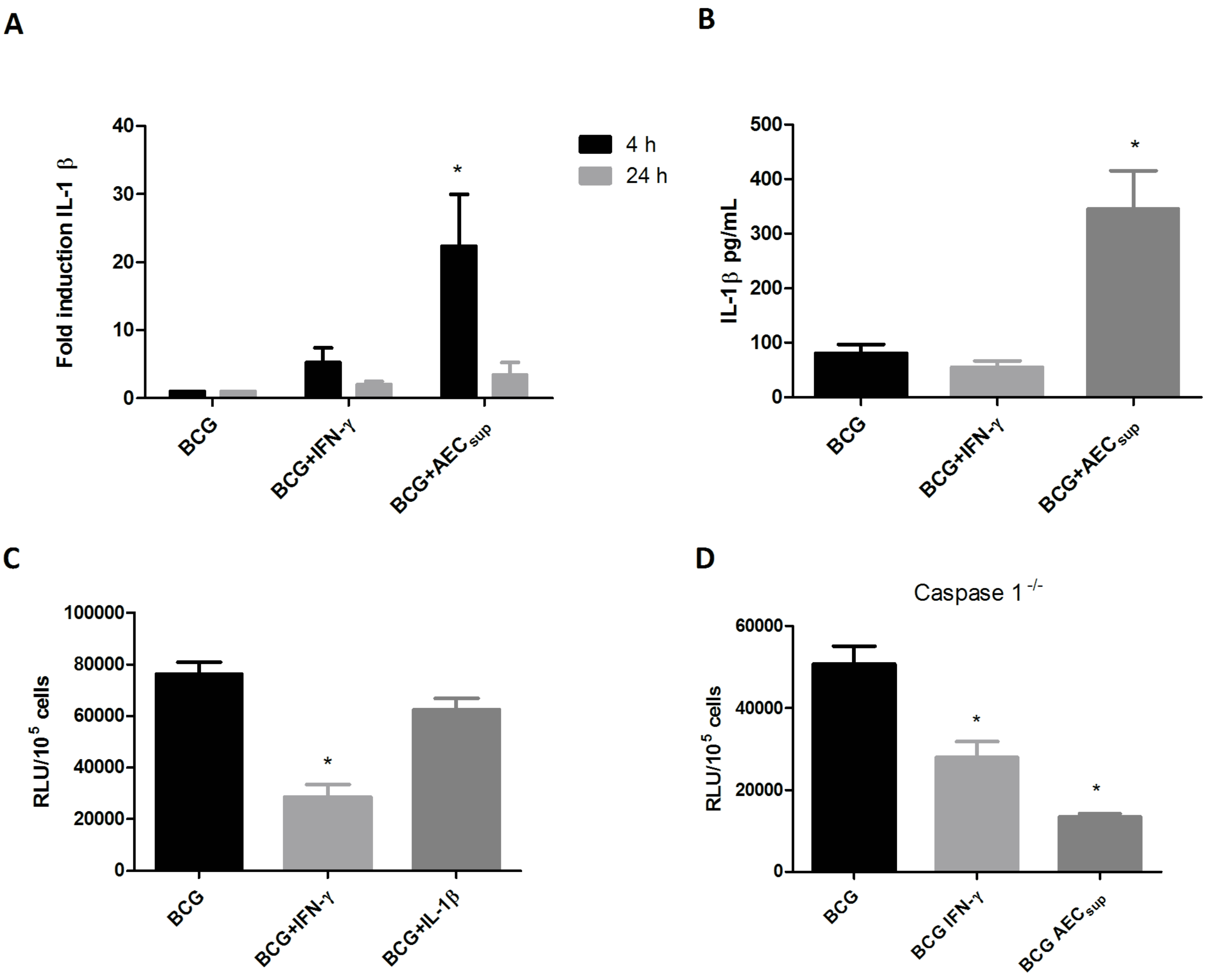

Figure 6. AEC_{sup} induces IL-1β expression and secretion without an effect of intracellular killing of BCG. BMM and PuM were infected with GFP-BCG. After infection, cells were treated with either IFN-γ or AEC_{sup} or left untreated. a) Total RNA was extracted from both cell-types after 4 and 24 h of treatment and the mean-fold accumulation of IL-1β transcripts shown. Data shows the average of 3 independent experiments. b) Cell culture supernatants were collected 24 h after infection and IL-1β measured with ELISA. Data shows the average of 6 independent experiments. c) The effect of incubating cells with IL-1β (1000 pg/ml) on intracellular growth of BCG. Data representative of 3 independent experiments d) The effect of $AECs_{up}$ on intracellular growth of BCG in BMM from caspase1$^{-/-}$ mice. Bacterial growth was evaluated by determining RLU. Values are expressed as means ± SD from 5 wells. The data is representative of 2 independent experiments. The differences between groups of BMM were analyzed using a one-way ANOVA followed by Bonferroni's Multiple Comparison Test. * significantly different from medium control, $P<0.05$.
doi:10.1371/journal.pone.0103411.g006

were involved in the un-responsiveness of PuM to IFN-γ by testing the response in IFN-γ$^{-/-}$SOCS1$^{-/-}$ mice. Interestingly, PuM from these mice responded to IFN-γ and efficiently controlled the intracellular bacteria. This showed not only that the transmission of IFN-γ signals was regulated by SOCS1 but again that PuM were capable of responding to IFN-γ.

Since iNOS and Arg-1 are co-regulated, we followed their induction on PuM and BMM after treatment with IFN-γ or AEC-derived media. AEC_{sup} induced Arg-1 on both cell-types but iNOS was only detected after treatment with IFN-γ. This lack of upregulation of iNOS expression, together with the lack of effect of NMMLA, a NOS inhibitor [47], all strongly indicate that mechanisms independent of the iNOS pathway were involved in the macrophage microbicidal activation by AEC factors. The profiles were also different regarding expression of SOCS1, only induced upon IFN-γ activation in both cell types. SOCS1 have been shown to be important in determining macrophage phenotype and function and regulate the balance between Arg-1 and iNOS activity. SOCS1 are also associated with the induction of Arg-1 in IL-4 treated macrophages [48]. Contrary to the IL-4-induced M2 macrophages described by Whyte et al. we show that AEC-derived factors induced Arg-1 in the absence of SOCS1 expression. Most important, AEC-derived factors promoted control of intracellular bacterial growth in PuM in a SOCS1 independent manner demonstrating that none of the factors secreted by AEC were involved in the induction of SOCS1 despite the fact that AEC-derived factors induced high expression of MMR and Arg-1 similar to M2-like macrophages [7].

To explore other possible mechanisms for the control of intracellular bacterial growth promoted by AEC factors, we looked at the formation of reactive oxygen species ROS and to IL-1β involved in autophagy related mechanisms. AEC factors were efficient inducers of IL-1β but, in contrast to what was reported by other groups [27], IL-1β was not responsible for the induced intracellular killing in our experimental setup. In BMM, the intracellular BCG growth was not affected by addition of exogenous IL-1β to the cultures. Moreover, BMM from caspase

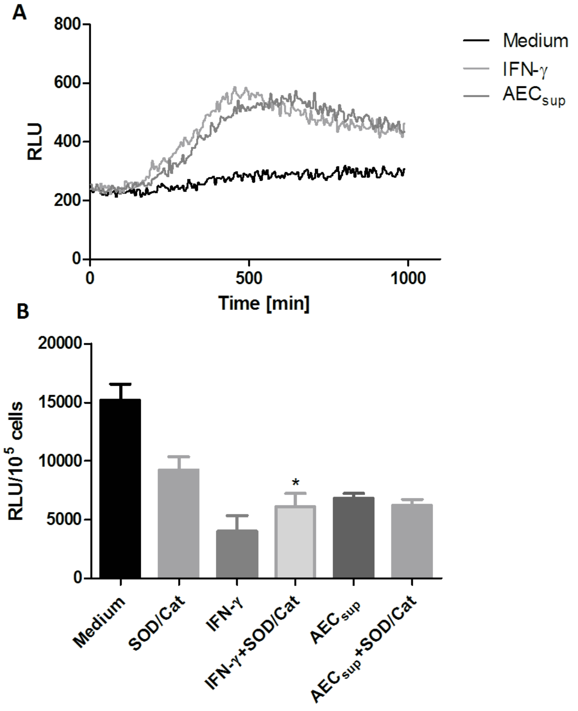

Figure 7. IFN-γ and AEC_{sup} induce an oxidative burst following infection with BCG. BMM were treated with IFN-γ (20 ng/ml) or AEC_{sup} 24 h prior to infection and then infected with GFP-BCG. a) ROS production was monitored in the cultures for 16 h by measuring luminescence with luminol as substrate. b) The effect of incubating cells with superoxide dismutase (SOD) and catalase on intracellular killing of BCG is shown. Values are expressed as means ± SD from 5 wells. Differences between treatments with or without SOD/catalase were analyzed using a paired t-test. * significant effect of SOD/catalase treatment, $P<0.05$.
doi:10.1371/journal.pone.0103411.g007

$1^{-/-}$ mice, unable to produce mature IL-1β, efficiently responded to stimulation by AEC factors.

ROS formation may also be important in the control of BCG infection through the oxidative burst [28]. To a similar extent as IFN-γ, AEC factors were efficient inducers of ROS but by themselves did not contribute to the control of intracellular BCG growth. Finally, we investigated a possible effect on the phagosomal compartment by treating BMM with chloroquine, an inhibitor of phagosomal acidification but we could not observe a major difference between the treated and the untreated groups.

We have been unable to directly ascribe the effect of AEC factors to the most common mechanisms operating separately. Thus, it may be that other mechanisms, not considered herein are responsible for these effects. An alternative explanation is that the combination of different microbicidal events and pathways could be operating in mycobacterial killing as described by Lefevre et al. where ROS contribute partially to IL-1β secretion in the responses to *L. infantum* [46].

Collectively, our data show that PuM are restricted in inflammatory responses mediated by IFN-γ through SOCS1 and that AEC-secreted factors enhance the microbicidal capacities of

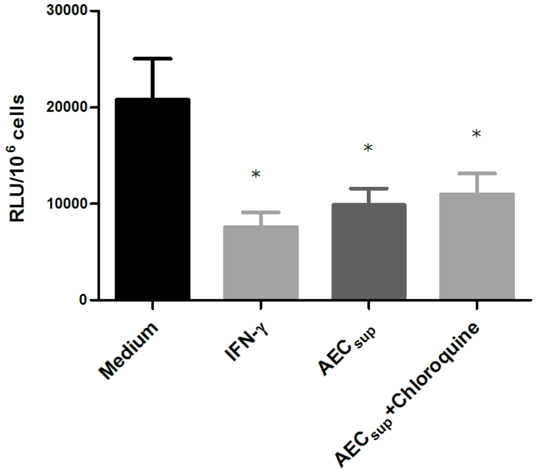

Figure 8. Inhibiting phagosomal acidification does not affect intracellular killing of BCG. BMM and PuM were infected with GFP-BCG. After infection, cells were treated with either IFN-γ or AEC_{sup} with or without chloroquine (10 μM). Bacterial growth was evaluated by determining RLU. Values are expressed as means ± SD from 5 wells. The data is representative of 2 independent experiments. Differences between treatments analyzed using a one-way ANOVA followed by Bonferroni's Multiple Comparison Test. * significantly different from medium control, $P<0.05$.
doi:10.1371/journal.pone.0103411.g008

macrophages by iNOS-independent mechanisms that may be linked with to an intermediate M1-M2-like macrophage.

Supporting Information

Figure S1 Effect of either pretreating PuM with IFN-γ before (IFN-γ Pre Inf), or after infection (IFN-γ Post Inf). PuM were either pretreated with IFN-γ (20 ng/ml) for 24 h before infection or left untreated. Cells were then infected with GFP-BCG for 4 h. After infection, cells were thoroughly washed and treated with gentamicin for 30 min. After additional washing, cells were either cultured in complete medium (Medium) or in medium with IFN-γ (20 ng/ml) or in medium with AEC-derived supernatant (AEC_{sup} 1:2 diluted) for 48 h. Bacterial growth was evaluated by determining RLU in cell lysates. Data are shown as RLU/10^6. Values are means ± SD of the mean from 2 independent experiments. Differences were analyzed with a one-way ANOVA. * significantly different from Medium.
(TIF)

Acknowledgments

The authors wish to thank Martin Rottenberg (MTC, Karolinska Institutet, Stockholm, Sweden) for supplying the IFN-$\gamma^{-/-}$SOCS$1^{-/-}$ mice and Sara Musciol and B. Heriques-Normark (MTC, Karolinska Institutet, Stockholm, Sweden) for supplying the Caspase-$1^{-/-}$ BMM.

Author Contributions

Conceived and designed the experiments: DHP ODC RF CF. Performed the experiments: DHP ODC RF. Analyzed the data: DHP ODC RF CF. Contributed reagents/materials/analysis tools: CF. Wrote the paper: DHP ODC RF CF.

References

1. Fehrenbach H (2012) Alveolar epithelial type II cells from embryonic stem cells: Knights in shining armour? Eur Respir J 39: 240–241.
2. Debbabi H, Ghosh S, Kamath AB, Alt J, Demello DE, et al. (2005) Primary type II alveolar epithelial cells present microbial antigens to antigen-specific CD4+ T cells. Am J Physiol Lung Cell Mol Physiol 289: L274–9.

3. Pechkovsky DV, Goldmann T, Ludwig C, Prasse A, Vollmer E, et al. (2005) CCR2 and CXCR3 agonistic chemokines are differently expressed and regulated in human alveolar epithelial cells type II. Respir Res 6: 75.
4. Lo B, Hansen S, Evans K, Heath JK, Wright JR (2008) Alveolar epithelial type II cells induce T cell tolerance to specific antigen. J Immunol 180: 881–888.
5. Witherden IR, Vanden Bon EJ, Goldstraw P, Ratcliffe C, Pastorino U, et al. (2004) Primary human alveolar type II epithelial cell chemokine release: Effects of cigarette smoke and neutrophil elastase. Am J Respir Cell Mol Biol 30: 500–509.
6. Thorley AJ, Ford PA, Giembycz MA, Goldstraw P, Young A, et al. (2007) Differential regulation of cytokine release and leukocyte migration by lipopolysaccharide-stimulated primary human lung alveolar type II epithelial cells and macrophages. J Immunol 178: 463–473.
7. Chuquimia OD, Petursdottir DH, Periolo N, Fernandez C (2013) Alveolar epithelial cells are critical in protection of the respiratory tract by secretion of factors able to modulate the activity of pulmonary macrophages and directly control bacterial growth. Infect Immun 81: 381–389.
8. Lawrence T, Natoli G (2011) Transcriptional regulation of macrophage polarization: Enabling diversity with identity. Nat Rev Immunol 11: 750–761.
9. Cassetta L, Cassol E, Poli G (2011) Macrophage polarization in health and disease. ScientificWorldJournal 11: 2391–2402.
10. Biswas SK, Chittezhath M, Shalova IN, Lim JY (2012) Macrophage polarization and plasticity in health and disease. Immunol Res 53: 11–24.
11. Martinez FO, Sica A, Mantovani A, Locati M (2008) Macrophage activation and polarization. Front Biosci 13: 453–461.
12. Vereyken EJ, Heijnen PD, Baron W, de Vries EH, Dijkstra CD, et al. (2011) Classically and alternatively activated bone marrow derived macrophages differ in cytoskeletal functions and migration towards specific CNS cell types. J Neuroinflammation 8: 58-2094-8-58.
13. Stein M, Keshav S, Harris N, Gordon S (1992) Interleukin 4 potently enhances murine macrophage mannose receptor activity: A marker of alternative immunologic macrophage activation. J Exp Med 176: 287–292.
14. Brysha M, Zhang JG, Bertolino P, Corbin JE, Alexander WS, et al. (2001) Suppressor of cytokine signaling-1 attenuates the duration of interferon gamma signal transduction in vitro and in vivo. J Biol Chem 276: 22086–22089.
15. Humphreys IR, Stewart GR, Turner DJ, Patel J, Karamanou D, et al. (2006) A role for dendritic cells in the dissemination of mycobacterial infection. Microbes Infect 8: 1339–1346.
16. Chuquimia OD, Petursdottir DH, Rahman MJ, Hartl K, Singh M, et al. (2012) The role of alveolar epithelial cells in initiating and shaping pulmonary immune responses: Communication between innate and adaptive immune systems. PLoS One 7: e32125.
17. Rahman MJ, Chuquimia OD, Petursdottir DH, Periolo N, Singh M, et al. (2011) Impact of toll-like receptor 2 deficiency on immune responses to mycobacterial antigens. Infect Immun 79: 4649–4656.
18. Corti M, Brody AR, Harrison JH (1996) Isolation and primary culture of murine alveolar type II cells. Am J Respir Cell Mol Biol 14: 309–315.
19. Carow B, Ye X, Gavier-Widen D, Bhuju S, Oehlmann W, et al. (2011) Silencing suppressor of cytokine signaling-1 (SOCS1) in macrophages improves mycobacterium tuberculosis control in an interferon-gamma (IFN-gamma)-dependent manner. J Biol Chem 286: 26873–26887.
20. Zhang X, Goncalves R, Mosser DM (2008) The isolation and characterization of murine macrophages. Curr Protoc Immunol Chapter 14: Unit 14.1.
21. Rothfuchs AG, Gigliotti D, Palmblad K, Andersson U, Wigzell H, et al. (2001) IFN-alpha beta-dependent, IFN-gamma secretion by bone marrow-derived macrophages controls an intracellular bacterial infection. J Immunol 167: 6453–6461.
22. Barrera LF, Kramnik I, Skamene E, Radzioch D (1994) Nitrite production by macrophages derived from BCG-resistant and -susceptible congenic mouse strains in response to IFN-gamma and infection with BCG. Immunology 82: 457–464.
23. Sato K, Akaki T, Tomioka H (1998) Differential potentiation of anti-mycobacterial activity and reactive nitrogen intermediate-producing ability of murine peritoneal macrophages activated by interferon-gamma (IFN-gamma) and tumour necrosis factor-alpha (TNF-alpha). Clin Exp Immunol 112: 63–68.
24. Flesch IE, Kaufmann SH (1991) Mechanisms involved in mycobacterial growth inhibition by gamma interferon-activated bone marrow macrophages: Role of reactive nitrogen intermediates. Infect Immun 59: 3213–3218.
25. Alexander WS, Starr R, Fenner JE, Scott CL, Handman E, et al. (1999) SOCS1 is a critical inhibitor of interferon gamma signaling and prevents the potentially fatal neonatal actions of this cytokine. Cell 98: 597–608.
26. Bronte V, Zanovello P (2005) Regulation of immune responses by L-arginine metabolism. Nat Rev Immunol 5: 641–654.
27. Pilli M, Arko-Mensah J, Ponpuak M, Roberts E, Master S, et al. (2012) TBK-1 promotes autophagy-mediated antimicrobial defense by controlling autophagosome maturation. Immunity 37: 223–234.
28. Ehrt S, Schnappinger D (2009) Mycobacterial survival strategies in the phagosome: Defence against host stresses. Cell Microbiol 11: 1170–1178.
29. Yu K, Mitchell C, Xing Y, Magliozzo RS, Bloom BR, et al. (1999) Toxicity of nitrogen oxides and related oxidants on mycobacteria: M. tuberculosis is resistant to peroxynitrite anion. Tuber Lung Dis 79: 191–198.
30. Gwinn MR, Vallyathan V (2006) Respiratory burst: Role in signal transduction in alveolar macrophages. J Toxicol Environ Health B Crit Rev 9: 27–39.
31. Alvarez E, Leiro J, Orallo F (2002) Effect of (-)-epigallocatechin-3-gallate on respiratory burst of rat macrophages. Int Immunopharmacol 2: 849–855.
32. Benoit M, Desnues B, Mege JL (2008) Macrophage polarization in bacterial infections. J Immunol 181: 3733–3739.
33. Taub DD, Key ML, Clark D, Turcovski-Corrales SM (1995) Chemotaxis of T lymphocytes on extracellular matrix proteins. analysis of the in vitro method to quantitate chemotaxis of human T cells. J Immunol Methods 184: 187–198.
34. Tschernig T, Pabst R (2009) What is the clinical relevance of different lung compartments? BMC Pulm Med 9: 39-2466-9-39.
35. Laskin DL, Weinberger B, Laskin JD (2001) Functional heterogeneity in liver and lung macrophages. J Leukoc Biol 70: 163–170.
36. Schneberger D, Aharonson-Raz K, Singh B (2011) Monocyte and macrophage heterogeneity and toll-like receptors in the lung. Cell Tissue Res 343: 97–106.
37. Steinmuller C, Franke-Ullmann G, Lohmann-Matthes ML, Emmendorffer A (2000) Local activation of nonspecific defense against a respiratory model infection by application of interferon-gamma: Comparison between rat alveolar and interstitial lung macrophages. Am J Respir Cell Mol Biol 22: 481–490.
38. Lohmann-Matthes ML, Steinmuller C, Franke-Ullmann G (1994) Pulmonary macrophages. Eur Respir J 7: 1678–1689.
39. Gordon SB, Read RC (2002) Macrophage defences against respiratory tract infections. Br Med Bull 61: 45–61.
40. Gordon S, Martinez FO (2010) Alternative activation of macrophages: Mechanism and functions. Immunity 32: 593–604.
41. Mantovani A, Locati M (2009) Orchestration of macrophage polarization. Blood 114: 3135–3136.
42. Anders HJ, Ryu M (2011) Renal microenvironments and macrophage phenotypes determine progression or resolution of renal inflammation and fibrosis. Kidney Int 80: 915–925.
43. Orme J, Mohan C (2012) Macrophage subpopulations in systemic lupus erythematosus. Discov Med 13: 151–158.
44. Edwards JP, Zhang X, Frauwirth KA, Mosser DM (2006) Biochemical and functional characterization of three activated macrophage populations. J Leukoc Biol 80: 1298–1307.
45. Sironi M, Martinez FO, D'Ambrosio D, Gattorno M, Polentarutti N, et al. (2006) Differential regulation of chemokine production by fcgamma receptor engagement in human monocytes: Association of CCL1 with a distinct form of M2 monocyte activation (M2b, type 2). J Leukoc Biol 80: 342–349.
46. Lefevre L, Lugo-Villarino G, Meunier E, Valentin A, Olagnier D, et al. (2013) The C-type lectin receptors dectin-1, MR, and SIGNR3 contribute both positively and negatively to the macrophage response to leishmania infantum. Immunity 38: 1038–1049.
47. Hibbs JB,Jr, Vavrin Z, Taintor RR (1987) L-arginine is required for expression of the activated macrophage effector mechanism causing selective metabolic inhibition in target cells. J Immunol 138: 550–565.
48. Whyte CS, Bishop ET, Ruckerl D, Gaspar-Pereira S, Barker RN, et al. (2011) Suppressor of cytokine signaling (SOCS)1 is a key determinant of differential macrophage activation and function. J Leukoc Biol 90: 845–854.
